# Closed-Loop Estimation of Retinal Network Sensitivity by Local Empirical Linearization

**DOI:** 10.1523/ENEURO.0166-17.2017

**Published:** 2018-01-23

**Authors:** Ulisse Ferrari, Christophe Gardella, Olivier Marre, Thierry Mora

**Affiliations:** 1 Institut de la Vision, Sorbonne Université, INSERM, CNRS, 17 rue Moreau, 75012 Paris, France; 2 Laboratoire de physique statistique, CNRS, Sorbonne Université, Université Paris-Diderot and École normale supérieure (PSL), 24, rue Lhomond, 75005 Paris, France

**Keywords:** Efficient coding theory, Sensory system, Retina, Closed-loop experiments, Fisher Information

## Abstract

Understanding how sensory systems process information depends crucially on identifying which features of the stimulus drive the response of sensory neurons, and which ones leave their response invariant. This task is made difficult by the many nonlinearities that shape sensory processing. Here, we present a novel perturbative approach to understand information processing by sensory neurons, where we linearize their collective response locally in stimulus space. We added small perturbations to reference stimuli and tested if they triggered visible changes in the responses, adapting their amplitude according to the previous responses with closed-loop experiments. We developed a local linear model that accurately predicts the sensitivity of the neural responses to these perturbations. Applying this approach to the rat retina, we estimated the optimal performance of a neural decoder and showed that the nonlinear sensitivity of the retina is consistent with an efficient encoding of stimulus information. Our approach can be used to characterize experimentally the sensitivity of neural systems to external stimuli locally, quantify experimentally the capacity of neural networks to encode sensory information, and relate their activity to behavior.

## Significance Statement

Understanding how sensory systems process information is an open challenge mostly because these systems have many unknown nonlinearities. A general approach to studying nonlinear systems is to expand their response perturbatively. Here, we apply such a method experimentally to understand how the retina processes visual stimuli. Starting from a reference stimulus, we tested whether small perturbations to that reference (chosen iteratively using closed-loop experiments) triggered visible changes in the retinal responses. We then inferred a local linear model to predict the sensitivity of the retina to these perturbations, and showed that this sensitivity supported an efficient encoding of the stimulus. Our approach is general and could be used in many sensory systems to characterize and understand their local sensitivity to stimuli.

## Introduction

An important issue in neuroscience is to understand how sensory systems use their neural resources to represent information. A crucial step toward understanding the sensory processing performed by a given brain area is to characterize its sensitivity (Benichoux et al., 2017), by determining which features of the sensory input are coded in the activity of these sensory neurons, and which features are discarded. If a sensory area extracts a given feature from the sensory scene, any change along that dimension will trigger a noticeable change in the activity of the sensory system. Conversely, if the information about a given feature is discarded by this area, the activity of the area should be left invariant by a change along that feature dimension. To understand which information is extracted by a sensory network, we must determine which changes in the stimulus evoke a significant change in the neural response, and which ones leave the response invariant.

This task is made difficult by the fact that sensory structures process stimuli in a highly nonlinear fashion. At the cortical level, many studies have shown that the response of sensory neurons is shaped by multiple nonlinearities (Machens et al., 2004; Carandini et al., 2005). Models based on the linear receptive field are not able to predict the responses of neurons to complex, natural scenes. This is even true in the retina. While spatially uniform or coarse grained stimuli produce responses that can be predicted by quasi-linear models (Berry and Meister, 1998; Keat et al., 2001; Pillow et al., 2008), stimuli closer to natural scenes (Heitman et al., 2016) or with rich temporal dynamics (Berry et al., 1999; Olveczky et al., 2003) are harder to characterize, as they trigger nonlinear responses in the retinal output. These unknown nonlinearities challenge our ability to model stimulus processing and limit our understanding of how neural networks process information.

Here, we present a novel approach to measure experimentally the local sensitivity of a nonlinear network. Because any nonlinear function can be linearized around a given point, we hypothesized that, even in a sensory network with nonlinear responses, one can still define experimentally a local linear model that can well predict the network response to small perturbations around a given reference stimulus. This local model should only be valid around the reference stimulus, but it is sufficient to predict if small perturbations can be discriminated based on the network response.

This local model allows us to estimate the sensitivity of the recorded network to changes around one stimulus. This local measure characterizes the ability of the network to code different dimensions of the stimulus space, circumventing the impractical task of building a complete accurate nonlinear model of the stimulus-response relationship. Although this characterization is necessarily local and does not generalize to the entire stimulus space, one can hope to use it to reveal general principles that are robust to the chosen reference stimulus.

We applied this strategy to the retina. We recorded the activity of a large population of retinal ganglion cells stimulated by a randomly moving bar. We characterized the sensitivity of the retinal population to small stimulus changes, by testing perturbations around a reference stimulus. Because the stimulus space is of high dimension, we designed closed-loop experiments to probe efficiently a perturbation space with many different shapes and amplitudes. This allowed us to build a complete model of the population response in that region of the stimulus space, and to precisely quantify the sensitivity of the neural representation.

We then used this experimental estimation of the network sensitivity to tackle two long-standing issues in sensory neuroscience. First, when trying to decode neural activity to predict the stimulus presented, it is always difficult to know if the decoder is optimal or if it misses some of the available information. We show that our estimation of the network sensitivity gives an upper bound of the decoder performance that should be reachable by an optimal decoder. Second, the efficient coding hypothesis (Attneave, 1954; Barlow, 1961) postulates that neural encoding of stimuli has adapted to represent natural occurring sensory scenes optimally in the presence of limited resources. Testing this hypothesis for sensory structures that perform nonlinear computations on high dimensional stimuli is still an open challenge. Here, we found that the network sensitivity with respect to stimulus perturbations exhibits a peak as a function of the temporal frequency of the perturbation, in agreement with prediction from efficient coding theory. Our method paves the way toward testing efficient coding theory in nonlinear networks.

## Materials and Methods

### Extracellular recording

Experiments were performed on the adult Long Evans rat of either sex, in accordance with institutional animal care standards. The retina was extracted from the euthanized animal and maintained in an oxygenated Ames’ medium (Sigma-Aldrich). The retina was recorded extracellularly on the ganglion cell side with an array of 252 electrodes spaced by 60 µm (Multichannel Systems), as previously described (Marre et al., 2012). Single cells were isolated offline using SpyKING CIRCUS a custom spike sorting algorithm (Yger et al., 2016). We then selected 60 cells that were well separated (no violations of refractory period, i.e., no spikes separated by <2 ms), had enough spikes (firing rate larger than 0.5 Hz), had a stable firing rate during the whole experiment, and responded consistently to repetitions of a reference stimulus (see Materials and Methods/Stimulus).

### Stimulus

The stimulus was a movie of a white bar on a dark background projected at a refresh rate of 50 Hz with a digital micromirror device. The bar had intensity 7.6 × 10^11^ photons/cm^– 2^/s^– 1^, and 115-µm width. The bar was horizontal and moved vertically. The bar trajectory consisted in 17034 snippets of 0.9 s consisting in two reference trajectories repeated 391 times each, perturbations of these reference trajectories and 6431 random trajectories. Continuity between snippets was ensured by constraining all snippets to start and end in the middle of the screen with velocity 0. Random trajectories followed the statistics of an overdamped stochastic oscillator (Deny et al., 2017). We used a Metropolis-Hastings algorithm to generate random trajectories satisfying the boundary conditions. The two reference trajectories were drawn from that ensemble.

### Perturbations

**Figure 1.**
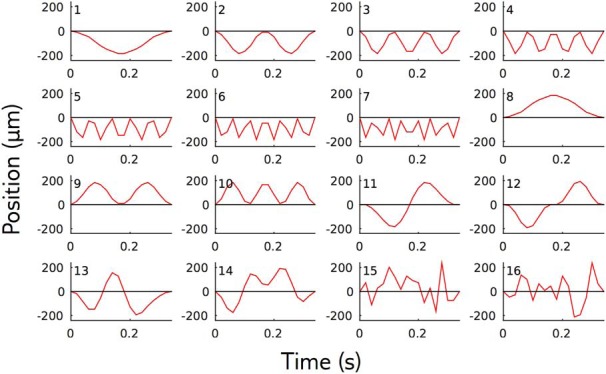
Perturbations shapes. We used the same 16 perturbation shapes for the two reference stimuli. The first 12 perturbation shapes were combinations of two Fourier components, and the last four ones were random combinations of them: $f_{k}(t)=\cos(2\pi kt/T)$ and $g_{k}(t)=(1/k)\;*\;\sin(2\pi t\;*\;k/T)$, with *T* the duration of the perturbation and *t* = 0 the beginning of the perturbation. The first perturbations *j* = 1…7 were $S_{j}=f_{j}-1$. For *j* = 8,…,10, they were the opposite of the three first ones: $S_{j}=-S_{j-7}$. For *j* = 11, 12 we used $S_{j}=g_{j-10+1}-g_{1}$. Perturbations 13 and 14 were random combinations of perturbations 1, 2, 3, 11, and 12, constrained to be orthogonal. Perturbations 15 and 16 were random combinations of *f_j_* for *j* ∈ [1,8] and *g_k_* for *k* ∈ [1,7], allowing higher frequencies than perturbation directions 13 and 14. Perturbation direction 15 and 16 were also constrained to be orthogonal. The largest amplitude for each perturbation we presented was 115 µm. An exception was made for perturbations 15 and 16 applied to the second reference trajectory, as for this amplitude they had a discrimination probability below 70%. They were thus increased by a factor 1.5. The largest amplitude for each perturbation was repeated at least 93 times, with the exception of perturbation 15 (32 times) and 16 (40 times) on the second reference trajectory.

Stimulus perturbations were small changes in the middle portion of the reference trajectory, between 280 and 600 ms. A perturbation is denoted by its discretized time series with time step *δt* = 20 ms, $S=(S_{1},\ldots ,S_{L})$, with *L* = 16, over the 320 ms of the perturbation (bold symbols represent vectors and matrices throughout). Perturbations can be decomposed as S=A×Q, where $A^{2}=(1/L)\overset{L}{\underset{t=1}{\sum }}S_{t}^{2}$ is the amplitude, and Q=S/A the shape. Perturbations shapes were chosen to have zero value and zero derivative at their boundaries (Fig. 1).

### Closed-loop experiments

We aimed to characterize the population discrimination capacity of small perturbations to the reference stimulus. For each perturbation shape (Fig. 1), we searched for the smallest amplitude that will still evoke a detectable change in the retinal response, as we explain below. To do this automatically on the many tested perturbation shapes, we implemented closed-loop experiments (Fig. 3*A*). At each iteration, the retina was stimulated with a perturbed stimulus and the population response was recorded and used to select the next stimulation in real time.

### Online spike detection

During the experiment we detected spikes in real time on each electrode independently. Each electrode signal was high-pass filtered using a Butterworth filter with a 200-Hz frequency cutoff. A spike was detected if the electrode potential *U* was lower than a threshold of five times the median absolute deviation of the voltage (Yger et al., 2016).

### Online adaptation of perturbation amplitude

To identify the range of perturbations that were neither too easy nor too hard to discriminate, we adapted perturbation amplitudes so that the linear discrimination probability (see below) converged to target value D* = 85%. For each shape, perturbation amplitudes were adapted using the Accelerated Stochastic Approximation (Kesten, 1958). If an amplitude *A_n_* triggered a response with discrimination probability *D_n_*, then at the next step the perturbation was presented at amplitude *A_n_*_+1_ with

(1) $$\ln A_{n+1}=\ln A_{n}-\frac{C}{r_{n}+1}(D_{n}-D^{*}),$$

where *C* = 0.74 is a scaling coefficient that controls the size of steps, and *r_n_* is the number of reversal steps in the experiment, i.e., the number of times when a discrimination *D_n_* larger than *D* * was followed by *D_n_*_+1_ smaller than *D* *, and vice versa. to explore the responses to different ranges of amplitudes even in the case where the algorithm converged too fast, we also presented amplitudes regularly spaced on a log-scale. We presented the largest amplitude *A*_max_ (Fig. 1, value), and scaled it down by multiples of 1.4, $A_{\text{max}}/1.4^{k}$ with *k* = 1,…,7.

### Online and offline linear discrimination

We applied linear discrimination theory to estimate if perturbed and reference stimuli can be discriminated from the population response they trigger. We applied it twice: online, on the electrode signals to adapt the perturbation amplitude, and offline, on the sorted spikes to estimate the response discrimination capacity. The response $R=(R_{ib})$ over time of either the *N* = 256 electrodes, or the *N* = 60 cells (the same notation *N* and **R** are used for electrode number and response and cell number and response for mathematical convenience), was binarized into *B* time bins of size *δ* = 20 ms: *R_ib_* = 1 if cell *i* spiked at least once during the *b*th time bin, and 0 otherwise. **R** is thus a vector of size *N* × *B*, labeled by a joint index *ib*. The response is considered from the start of the perturbation until 280 ms after its end, so that *B* = 30.

To apply linear discrimination on $R_{S}$, the response to the perturbation **S**, we record multiple responses $R_{\text{ref}}$ to the reference, and multiple responses $R_{S_{\text{max}}}$ to a large perturbation $S_{\text{max}}$, with the same stimulus shape as **S** but at the maximum amplitude that was played during the course of the experiment (typically 110 μm; Fig. 1). Our goal is to estimate how close $R_{S}$ is to the “typical” $R_{\text{ref}}$ compared to the typical $R_{S_{\text{max}}}$. To this aim, we compute the mean response to the reference and to the large perturbation, $〈R_{\text{ref}}〉$ and $〈R_{S_{\text{max}}}〉$, and use their difference as a linear classifier. Specifically, we project $R_{S}$ onto the difference between these two mean responses. For a generic response R (either $R_{\text{ref}},\;R_{S}$ or $R_{S_{\text{max}}}$), the projection *x* (respectively, $x_{\text{ref}},\;x_{S}$ or $x_{S_{\text{max}}}$) reads:

(2) $$x=u^{\text{T}}\;\cdot \;R$$

where *x* is a scalar and $u=〈R_{S_{\text{max}}}〉-〈R_{\text{ref}}〉$ is the linear discrimination axis. The computation of *x* is a projection in our joint index notation, but it can be decomposed in a summation over cells *i* and consecutive time-bins *b* of the response: $x=\sum_{i}\sum_{b}u_{ib}R_{ib}$. On average, we expect $〈x_{\text{ref}}〉<〈x_{S}〉<〈x_{S_{\text{max}}}〉$. To quantify the discrimination capacity, we compute the probability that $x_{S}>x_{\text{ref}}$, following the classical approach for linear classifiers.

To avoid overfitting, when projecting a response to the reference trajectory, $R_{\text{ref}}$, onto $\left(〈R_{S_{\text{max}}}〉-〈R_{\text{ref}}〉\right)$, we first re-compute $〈R_{\text{ref}}〉$ by leaving out the response of interest. If we did not do this, the discriminability of responses would be overestimated.

In Discussion, Mathematical derivations, we discuss the case of a system with response changes that are linear in the perturbation, or equivalently when the perturbation is small enough so that a linear first order approximation is valid.

### Offline discrimination and sensitivity

To measure the discrimination probability as a function of the perturbation amplitude, we consider the difference of the projections, $\Delta x=x_{S}-x_{\text{ref}}$. The response to the stimulation $R_{S}$ is noisy, making *x* and *x*_ref_ the sum of many random variables (corresponding to each neuron and time bin combinations), and we can apply the central limit theorem to approximate their distributions as Gaussian (Fig. 3*B*, right side), for a given perturbation at a given amplitude. For small perturbations, the mean of Δ*x* grows linearly with the perturbation amplitude *A*, *μ* = *α* × *A*, and the variances of $x_{S}$ and $x_{\text{ref}}$ are equal at first order, $\text{Var}(x_{S})\approx \text{Var}(x_{\text{ref}})=\sigma^{2}$, so that the variance of Δ*x*, $\text{Var}(\Delta x)=\text{Var}(x_{S})+\text{Var}(x_{\text{ref}})=2\sigma^{2}$ is independent of *A*. Then the probability of discrimination is given by the error function:

(3) $$D=P(x_{\text{ref}}<x_{S})=\frac{1}{2}\left(1+\text{erf}(d\prime /2)\right)$$

where d′=μ/σ=c×A is the standard sensitivity index (Macmillan and Creelman, 2004), and *c* = *α*∕*σ* is defined as the sensitivity coefficient, which depends on the perturbation shape **Q**. This coefficient determines the amplitude *A* = *c* ^– 1^ at which discrimination probability is equal to (1/2)[1+erf(1/2)]=76%.

### Optimal sensitivity and Fisher information

We then aimed to find the discrimination probability for any perturbation. Given the distributions of responses to the reference stimulus, P(R|ref), and to a perturbation, P(R|S), optimal discrimination can be achieved by studying the sign of the response-specific log-ratio L(R)=ln[P(R|S)/P(R|ref)]. Note that in the log-ratio, **R** represents a stochastic response and not the independent variable of a probability density. Because it depends on the response **R**, this log ratio is both stimulus dependent and stochastic. Let us define $L_{\text{ref}}$ to be the random variable taking value L(R) on presentation of the reference stimulus, i.e., when **R** is a (stochastic) response to the stimulus, and $L_{S}$ the random variable taking value L(R) when **R** is a response to the presentation of **S**. According to the definition given earlier, the probability of successful discrimination is the probability that the log-ratio calculated from a random response to the perturbed stimulus is larger than the log-ratio calculated from a random response to the reference, $L_{S}>L_{\text{ref}}$. Using the central limit theorem, we assume again that $L_{S}$ and $L_{\text{ref}}$ are Gaussian. We can calculate their mean and variance at small **S** (see Discussion, Mathematical derivations): $\mu_{L}=〈L_{S}〉-〈L_{\text{ref}}〉=S^{\text{T}}\cdot I\cdot S$ and $2\sigma_{L}^{2}=\text{Var}(L_{S})+\text{Var}(L_{\text{ref}})=2S^{\text{T}}\cdot I\cdot S$, where

(4) $$I=(I_{tt\prime }),\;I_{tt\prime }=-\underset{R}{\sum }P(R|\text{ref}){\frac{\partial^{2}\ln P(R|S)}{\partial S_{t}\partial S_{t\prime }}|}_{S=0}$$

is the Fisher information matrix calculated at the reference stimulus. Following standard discrimination theory (Macmillan and Creelman, 2004; for a derivation in a similar context, see Seung and Sompolinsky, 1993), the discrimination probability is (see Discussion, Mathematical derivations): $D=P(L_{S}>L_{\text{ref}})=(1/2)[1+\text{erf}(d\prime /2)]$, with

(5) $$d\prime =\frac{\mu_{L}}{\sigma_{L}}=\sqrt{S^{\text{T}}\cdot I\cdot S}.$$

This result generalizes to an arbitrary stimulus dimension the result of Seung and Sompolinsky (1993).

### Local model

Because sampling the full response probability distribution P(R|S) would require estimating 2*^N^* ^×^*^B^* numbers (one for each possible response **R**) for each perturbation **S**, estimating the Fisher Information Matrix directly is impractical, and requires building a model that can predict how the retina responds to small perturbations of the reference stimulus. We used the data from these closed loop experiments for this purpose. The model, schematized in Figure 4*A*, assumes that a linear correction can account for the response change driven by small perturbations. We introduce the local model as a linear expansion of the logarithm of response distribution as a function of both stimulus and response:

(6) $$\ln\;P(R|S)=\ln\;P(R|ref)+\underset{i}{\sum }\underset{\left\{t_{i}\right\}}{\sum }\int \mathrm{dt}F_{i}(t_{i},t)S(t)+const=\ln\;P(R|ref)+\underset{ib,t}{\sum }R_{ib}F_{ib,t}S_{t}+const=\ln\;P(R|ref)+R^{T}\cdot F\cdot S+const,$$

where in the integral form, {*t_i_*} denotes the set of spiking times of neuron *i*, and *F_i_* is a stimulus filter depending on both the stimulus time and spiking time (no time-translation invariance). The second line is the discretized version adapted to our binary convention for describing spiking activity binned into bins indexed by *b*. The matrix $F=(F_{ib,t})$ is the discretized version of $F_{i}(t_{i},t)$ and contains the linear filters with which the change in the response is calculated from the linear projection of the past stimulus. For ease of notation, hereafter we use matrix multiplications rather than explicit sums over *ib* and *t*.

The distribution of responses to the reference trajectory is assumed to be conditionally independent:

(7) $$\ln P(R|\text{ref})=\sum_{ib}\ln P(R_{ib}|\text{ref}).$$

Since the variables *R_ib_* are binary, their mean values ⟨*R_ib_*⟩ on presentation of the reference completely specify $P(R_{ib}|\text{ref})$: $〈R_{ib}〉=P(R_{ib}=1|\text{ref})$. They are directly evaluated from the responses to repetitions of the reference stimulus, with a small pseudo-count to avoid zero values.

Evaluating the Fisher information matrix (Eq. 4), within the local model (Eq. 6), gives:

(8) $$I=F^{\text{T}}\cdot C_{R}\cdot F$$

where $C_{R}$ is the covariance matrix of **R**, which within the model is diagonal because of the assumption of conditional independence.

### Inference of the local model

To infer the filters *F_ib_*_,_*_t_*, we only include perturbations that are small enough to remain within the linear approximation. We first separated the dataset into a training (285 × 16 perturbations) and testing (20 × 16 perturbations) sets. We then defined, for each perturbation shape, a maximum perturbation amplitude above which the linear approximation was no longer considered valid. We selected this threshold by optimizing the model’s ability to predict the changes in firing rates in the testing set. Model learning was performed for each cell independently by maximum likelihood with an *L*_2_ smoothness regularization on the shape of the filters, using a pseudo-Newton algorithm. The amplitude threshold obtained from the optimization varied widely across perturbation shapes. The number of perturbations for each shape used in the inference ranged from 20 (7% of the total) to 260 (91% of the total). Overall only 32% of the perturbations were kept (as we excluded repetitions of perturbations with largest amplitude used for calibration). Overfitting was limited: when tested on perturbations of similar amplitudes, the prediction performance on the testing set was never lower than 15% of the performance on the training set.

### Linear decoder

We built a linear decoder of the bar trajectory from the population response. The model takes as input the population response **R** to the trajectory *X*(*t*) and provides a prediction $\hat{X}(t)$ of the bar position in time:

(9) $$\hat{X}(t)=\sum_{i,\tau }K_{i,\tau }R_{i,t-\tau }+\text{constant}$$

where the filters *K* have a time integration windows of 15 × 20 ms = 300 ms, as in the local model.

We inferred the linear decoder filters by minimizing the mean square error (Warland et al., 1997), $\sum_{t}[X(t)-\hat{X}(t){]}^{2}$, in the reconstruction of 4000 random trajectories governed by the dynamics of an overdamped oscillator with noise (see Materials and Methods/Stimulus). The linear decoder is then applied to the perturbed trajectories, *X*(*t*) = *X*_0_(*t*) + *S*(*t*), where *X*_0_(*t*) denotes the reference trajectory. The linear decoder does not use prior information about the local structure of the experiment, namely about the fact that the stimulus to decode consists of perturbations around a reference simulation. However, it implicitly uses prior information about the statistics of the overdamped oscillator, as it was trained on bar trajectories with those statistics. Tested on a sequence of ∼400 repetitions of one of the two reference trajectories, where the first 300 ms of each have been cut out, we obtain a correlation coefficient of 0.87 between the stimulus and its reconstruction.

### Local model Bayesian decoder

To construct a decoder based on the local model, we use Bayes’ rule to infer the presented stimulus given the response:

(10) $$P(S|R)=\frac{P(R|S)P(S)}{P(R)}$$

where P(R|S) is given by the local model (Eq. 6), P(S) is the prior distribution over the stimulus, and P(R) is the prior distribution over the stimulus, and P(R) is a normalization factor that does not depend on the stimulus. P(S) is taken to be the distribution of trajectories from an overdamped stochastic oscillator with the same parameters as in the experiment (Deny et al., 2017), to allow for a fair comparison with the linear decoder, which was trained with those statistics. The stimulus is inferred by maximizing the posterior P(S|R) numerically, using a pseudo-Newton iterative algorithm.

### Local signal to noise ratio in decoding

To quantify local decoder performance as a function of the stimulus frequency, we estimated a local signal-to-noise ratio (LSNR) of the decoding signal, LSNR(**S**), which is a function of the reference stimulus. Here, we cannot compute SNR as a ratio between total signal power and noise power, because this would require to integrate over the entire stimulus space, while our approach only provides a model around the neighborhood of the reference stimulus.

To obtain a meaningful comparison between the linear and local decoders, we expand them at first order in the stimulus perturbation and compute the SNR of these “linearized” decoders. For any decoder and for stimuli nearby a reference stimulation, the inferred value of the stimulus, X^, can be written as X^=ϕ(X), where **X** is the real bar trajectory, and ϕ has a random component (due to the random nature of the response on which the reconstruction relies). Linearizing ϕ for $X=X_{0}+S$,

(11) X^=ϕ(X0+S)≈〈ϕ(X0)〉+T·S+ϵ,

where **T** is a transfer matrix which differs from the identity matrix when decoding is imperfect, and ϵ a Gaussian noise of covariance $C_{\epsilon }$. Thus, the reconstructed perturbation S^=X^−X0 can be written as:

(12) S^=T·S+b+ϵ,

where $b=〈\phi (X_{0})〉-X_{0}$ is a systematic bias. We inferred the values of **b** and $C_{\epsilon }$ from the ∼400 reconstructions of the reference stimulation using either of the two decoders, and the values of **T** from the reconstructions of the perturbed trajectories. The inference is done by an iterative algorithm similar to that used for the inference of the filters **F** of the local model. We define the LSNR in decoding the perturbation **S** as:

(13) LSNR(S)=(〈S^〉−b)T·Cϵ−1·(〈S^〉−b)=ST·TT·Cϵ−1·T·S.

where here 〈…〉 means average with respect to the noise ϵ. In this formula, the signal is defined as the average predicted perturbation 〈S^〉, from which the systematic bias **b** is subtracted, yielding T·S. The noise is simply ϵ. Note that here the LSNR is defined for a given perturbation **S**. It is the ratio of the squared signal to the noise variance (summed over the eigendirections of the noise correlator, since we are dealing with a multidimensional signal). This LSNR gives a measure of decoding performance, through the amplitude of the decoded signal relative to the noise. To study how this performance depends on the frequency *ν* of the input signal, in Figure 6*C*, we apply Equation 13 with $S_{b}=A\exp(2\pi i\nu b\delta t)$, where *A* is the amplitude of the perturbation (Fig. 5*A*), and *b* is a time-bin counter. Note that this frequency-dependent LSNR should not be intepreted as a ratio of signal and noise power spectra, but rather as the dependence of decoding performance on the frequency of the perturbation. It is used rather than the traditional SNR because we are dealing with signals with no time-translation invariance (i.e., $T_{tt\prime }$ is not just a function of t−t′, and neither is $C_{\epsilon ,tt\prime }$). However, our LNSR reduces to the traditional frequency-dependent SNR in the special case of time-translation invariance, i.e., when the decoder is convolutional, and its noise stationary (see Discussion, Mathematical derivations)

### Fisher information estimation of sensitivity coefficients

In Figures 5*A*,*B*, 7*C*,*D*, we show the Fisher estimations of sensitivity coefficients c(Q) for perturbations of different shapes **Q**, either those used during the experiment (Fig. 1), or oscillating ones, $S_{b}=A\exp(2\pi i\nu b\delta t)$. to compute these sensitivity coefficients, we use Equation 14 to compute the sensitivity index d′ and then we divide it by the perturbation amplitude, yielding $c(Q)=d\prime /A=\sqrt{Q^{\text{T}}\cdot I\cdot Q}$.

## Results

### Measuring sensitivity using closed-loop experiments

**Figure 2.**
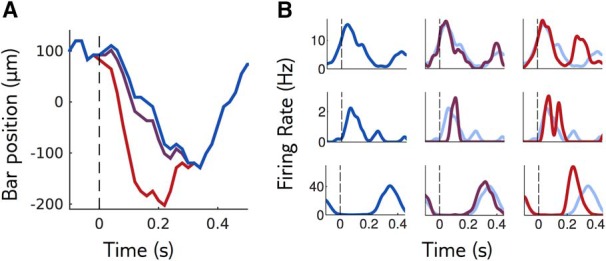
Sensitivity of a neural population to visual stimuli. ***A***, The retina is stimulated with repetitions of a reference stimulus (here the trajectory of a bar, in blue), and with perturbations of this reference stimulus of different shapes and amplitudes. Purple and red trajectories are perturbations with the same shape, of small and large amplitude. ***B***, Mean response of three example cells to the reference stimulus (left column and light blue in middle and right columns) and to perturbations of small and large amplitudes (middle and right columns).

We recorded from a population of 60 ganglion cells in the rat retina using a 252-electrode array while presenting a randomly moving bar (Fig. 2*A*; Materials and Methods). Tracking the position of moving objects is major task that the visual system needs to solve. The performance in this task is constrained by the ability to discriminate different trajectories from the retinal activity. Our aim was to measure how this recorded retinal population responded to different small perturbations around a pre-defined stimulus. We measured the response to many repetitions of a short (0.9 s) reference stimulus, as well as many small perturbations around it. The reference stimulus was the random trajectory of a white bar on a dark background undergoing Brownian motion with a restoring force (see Materials and Methods). Perturbations were small changes affecting that reference trajectory in its middle portion, between 280 and 600 ms. The population response was defined as sequences of spikes and silences in 20-ms time bins for each neuron, independently of the number of spikes (see Materials and Methods).

To assess the sensitivity of the retinal network, we asked how well different perturbations could be discriminated from the reference stimulus based on the population response. We expect the ability to discriminate perturbations to depend on two factors. First, the direction of the perturbation in the stimulus space, called perturbation shape. If we change the reference stimulus by moving along a dimension that is not taken into account by the recorded neurons, we should not see any change in the response. Conversely, if we choose to change the stimulus along a dimension that neurons “care about,” we should quickly see a change in the response. The second factor is the amplitude of the perturbation: responses to small perturbations should be hardly distinguishable, while large perturbations should elicit easily detectable changes (Fig. 2*B*). To assess the sensitivity to perturbations of the reference stimulus we need to explore many possible directions that these perturbations can take, and for each direction, we need to find a range of amplitudes that is as small as possible but will still evoke a detectable change in the retinal response. In other words, we need to find the range of amplitudes for which discrimination is hard but not impossible. This requires looking for the adequate range of perturbation amplitudes “online,” during the time course of the experiment.

**Figure 3.**
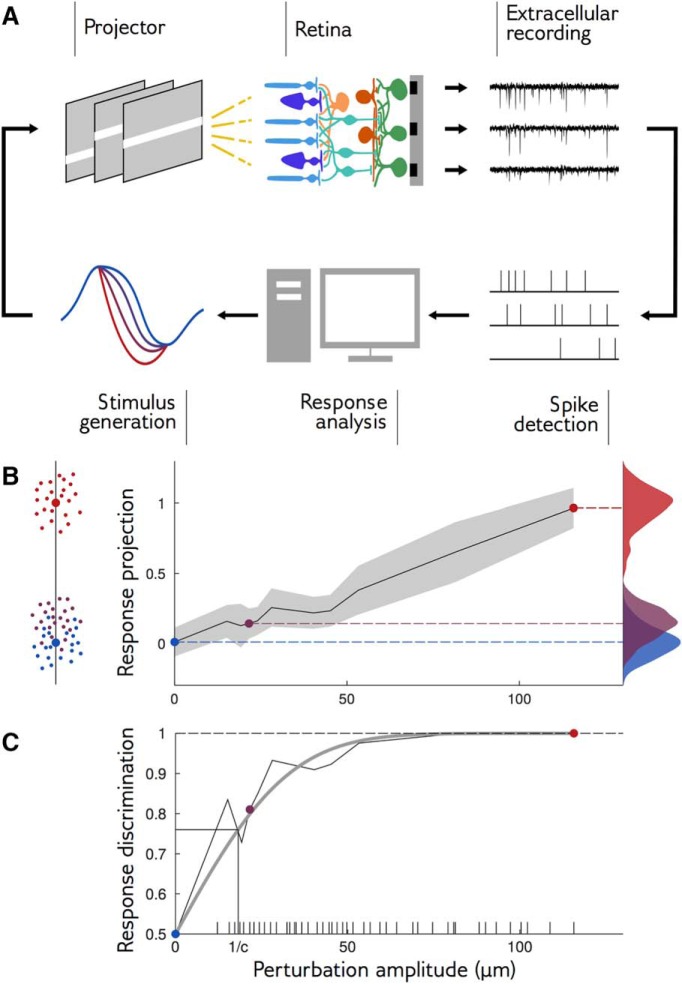
Closed-loop experiments to probe the range of stimulus sensitivity. ***A***, Experimental setup: we stimulated a rat retina with a moving bar. Retinal ganglion cell (RGC) population responses were recorded extracellularly with a multielectrode array. Electrode signals were high-pass filtered and spikes were detected by threshold crossing. We computed the discrimination probability of the population response and adapted the amplitude of the next perturbation. ***B***, left, The neural responses of 60 sorted RGCs are projected along the axis going through the mean response to reference stimulus and the mean response to a large perturbation. Small dots are individual responses, large dots are means. Middle, Mean and standard deviation (in gray) of response projections for different amplitudes of an example perturbation shape. Right, Distributions of the projected responses to the reference (blue), and to small (purple) and large (red) perturbations. Discrimination is high when the distribution of the perturbation is well separated from the distribution of the reference. ***C***, Discrimination probability as a function of amplitude *A*. The discrimination increases as an error function, (1/2)[1+erf(d′/2)], with d′=c×A (gray line: fit). Ticks on the *x*-axis show the amplitudes that have been tested during the closed-loop experiment.

To automatically adapt the amplitude of perturbations to the sensitivity of responses for each of the 16 perturbation shapes and for each reference stimulus, we implemented closed-loop experiments (Fig. 3*A*). At each step, the retina was stimulated with a perturbed stimulus and the population response was recorded. Spikes were detected in real time for each electrode independently by threshold crossing (see Materials and Methods). This coarse characterization of the response is no substitute for spike sorting, but it is fast enough to be implemented in real time between two stimulus presentations, and sufficient to detect changes in the response. This method was used to adaptively select the range of perturbations in real time during the experiment, and to do it for each direction of the stimulus space independently. Proper spike sorting was performed after the experiment using the procedure described in Marre et al., 2012 and Yger et al., (2016) and used for all subsequent analyses.

To test whether a perturbation was detectable from the retinal response, we considered the population response, summarized by a binary vector containing the spiking status of each recorded neuron in each time bin, and projected it onto an axis to obtain a single scalar number. The projection axis was chosen to be the difference between the mean response to a large-amplitude perturbation and the mean response to the reference (Fig. 3*B*). On average, the projected response to a perturbation is larger than the projected response to the reference. However, this may not hold for individual responses, which are noisy and broadly distributed around their mean (for example distributions, see Fig. 3*B*, right). We define the discrimination probability as the probability that the projected response to the perturbation is in fact larger than to the reference. Its value is 100% if the responses to the reference and perturbation are perfectly separable, and 50% if their distributions are identical, in which case the classifier does no better than chance. This discrimination probability is equal to the “area under the curve of the receiver-operating characteristics,” which is widely used for measuring the performance of binary discrimination tasks.

During our closed-loop experiment, our purpose was to find the perturbation amplitude with a discrimination probability of 85%. To this end, we computed the discrimination probability online as described above, and then chose the next perturbation amplitude to be displayed using the “accelerated stochastic approximation” method (Kesten, 1958; Faes et al., 2007): when discrimination was above 85%, the amplitude was decreased, otherwise, it was increased (see Materials and Methods).

Figure 3*C* shows the discrimination probability as a function of the perturbation amplitude for an example perturbation shape. Discrimination grows linearly with small perturbations, and then saturates to 100% for large ones. This behavior is well approximated by an error function (gray line) parametrized by a single coefficient, which we call sensitivity coefficient and denote by *c*. This coefficient measures how fast the discrimination probability increases with perturbation amplitude: the higher the sensitivity coefficient, the easier it is to discriminate responses to small perturbations. It can be interpreted as the inverse of the amplitude at which discrimination reaches 76%, and is related to the classical sensitivity index d′ (Macmillan and Creelman, 2004), through d′=c×A, where *A* denotes the perturbation amplitude (see Materials and Methods).

All 16 different perturbation shapes were displayed, corresponding to 16 different directions in the stimulus space, and the optimal amplitude was searched for each of them independently. We found a mean sensitivity coefficient of *c* = 0.0516 μm^– 1^. However, there were large differences across the different perturbation shapes, with a minimum of *c* = 0.028 μm^– 1^ and a maximum of *c* = 0.065 μm^– 1^.

### Sensitivity and Fisher information

So far, our results have allowed us to estimate the sensitivity of the retina in specific directions of the perturbation space. Can we generalize from these measurements and predict the sensitivity in any direction? The stimulus is the trajectory of a bar and is high dimensional. Under the assumptions of the central limit theorem, we show that the sensitivity can be expressed in matrix form as (see Materials and Methods):

(14) $$d\prime =\sqrt{S^{\text{T}}\cdot I\cdot S},$$

where **I** is the Fisher information matrix, of the same dimension as the stimulus, and **S** the perturbation represented as a column vector. This result generalizes that of Seung and Sompolinsky (1993), initially derived for one-dimensional stimuli, to arbitrary dimensions. Thus, the Fisher information is sufficient to predict the code’s sensitivity to any perturbation.

Despite the generality of Equation 14, it should be noted that estimating the Fisher information matrix for a highly dimensional stimulus ensemble requires a model of the population response. As already discussed in the introduction, the nonlinearities of the retinal code make the construction of a generic model of responses to arbitrary stimuli a very arduous task, and is still an open problem. However, the Fisher information matrix need only be evaluated locally, around the response to the reference stimulus, and to do so building a local response model is sufficient.

### Local model for predicting sensitivity

We introduce a local model to describe the stochastic population response to small perturbations of the reference stimulus. This model will then be used to estimate the Fisher information matrix, and from it the retina’s sensitivity to any perturbation, using Equation 14.

**Figure 4.**
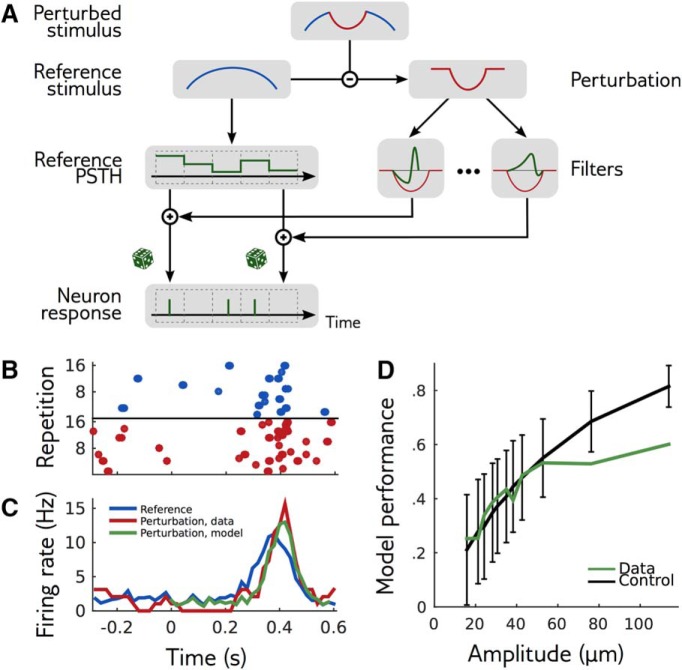
Local model for responses to perturbations. ***A***, The firing rates in response to a perturbation of a reference stimulus are modulated by filters applied to the perturbation. There is a different filter for each cell and each time bin. Because the model is conditionally independent across neurons we show the schema for one example neuron only. ***B***, Raster plot of the responses of an example cell to the reference (blue) and perturbed (red) stimuli for several repetitions. ***C***, PSTH of the same cell in response to the same reference (blue) and perturbation (red). Prediction of the local model for the perturbation is shown in green. ***D***, Performance of the local model at predicting the change in PSTH induced by a perturbation, as measured by Pearson correlation coefficient between data and model, averaged over cells (green). The data PSTH were calculated by grouping perturbations of the same shape and of increasing amplitudes by groups of 20 and computing the mean firing rate at each time over the 20 perturbations of each group. The model PSTH was calculated by mimicking the same procedure. To control for noise from limited sampling, the same performance was calculated from synthetic data of the same size, where the model is known to be exact (black).

The model, schematized in Figure 4*A*, assumes that perturbations are small enough that the response can be linearized around the reference stimulus. First, the response to the reference is described by conditionally independent neurons firing with time-dependent rates estimated from the peristimulus time histograms (PSTHs). Second, the response to perturbations is modeled as follows: for each neuron and for each 20-ms time bin of the considered response, we use a linear projection of the perturbation trajectory onto a temporal filter to modify the spike rates relative to the reference. These temporal filters were inferred from the responses to all the presented perturbations, varying both in shape and amplitude (but small enough to remain within the linear approximation). Details of the model and its inference are given in Materials and Methods.

We checked the validity of the local model by testing its ability to predict the PSTH of cells in response to perturbations (Fig. 4*B*). To assess model performance, we computed the difference of PSTH between perturbation and reference, and compared it to the model prediction. Figure 4*D* shows the correlation coefficient of this PSTH difference between model and data, averaged over all recorded cells for one perturbation shape. To obtain an upper bound on the attainable performance given the limited amount of data, we computed the same quantity for responses generated by the model (black line). Model performance saturates that bound for amplitudes up to 60 μm, indicating that the local model can accurately predict the statistics of responses to perturbations within that range. For larger amplitudes, the linear approximation breaks down, and the local model fails to accurately predict the response. This failure for large amplitudes is expected if the retinal population responds nonlinearly to the stimulus. We observed the same behavior for all the perturbation shapes that we tested. We have therefore obtained a local model that can predict the response to small enough perturbations in many directions.

**Figure 5.**
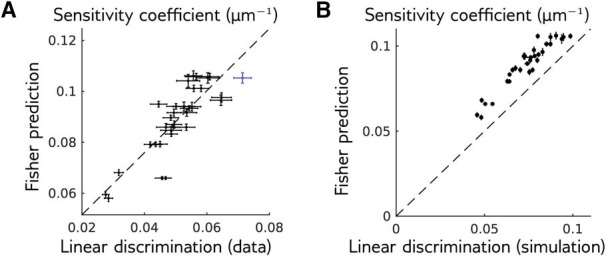
The Fisher information predicts the experimentally measured sensitivity. ***A***, Sensitivity coefficients *c* for the two reference stimuli and 16 perturbation shapes, measured empirically and predicted by the Fisher information (Eq. 14) and the local model. The purple point corresponds to the perturbation shown in Figure 2. Dashed line stands for best linear fit. ***B***, Same as ***A***, but for responses simulated with the local model, with the same amount of data as in experiments. The discriminability of perturbations was measured in the same way than for recorded responses. Dots and error bars stand for mean and SEM over 10 simulations. Dashed line stands for identity.

To further validate the local model, we combine it with Equation 14 to predict the sensitivity *c* of the network to various perturbations of the bar trajectory, as measured directly by linear discrimination (Fig. 3). The Fisher matrix takes a simple form in the local model: $I=F\cdot C_{R}\cdot F^{\text{T}}$, where **F** is the matrix containing the model’s temporal filters (stacked as row vectors), and $C_{R}$ is the covariance matrix of the entire response to the reference stimulus across neurons and time. We can then use the Fisher matrix to predict the sensitivity coefficient using Equation 14, and compare it to the same sensitivity coefficient previously estimated using linear discrimination. Figure 5*A* shows that these two quantities are strongly correlated (Pearson correlation: 0.82, *p* = 10^–8^), although the Fisher prediction is always larger. This difference could be due to two reasons: limited sampling of the responses, or nonoptimality of the projection axis used for linear discrimination. To evaluate the effect of finite sampling, we repeated the analysis on a synthetic dataset generated using the local model, with the same stimulation protocol as in the actual experiment. The difference in the synthetic data (Fig. 5*B*) and experiment (Fig. 5*A*) were consistent, suggesting that finite sampling is indeed the main source of discrepancy. We confirmed this result by checking that using the optimal discrimination axis (see Discussion, Mathematical derivations) did not improve performance (data not shown).

Summarizing, our estimation of the local model and of the Fisher information matrix can predict the sensitivity of the retinal response to perturbations in many directions of the stimulus space. We now use this estimation of the sensitivity of the retinal response to tackle two important issues in neural coding: the performance of linear decoding and efficient information transmission.

### Linear decoding is not optimal

When trying to decode the position of random bar trajectories over time using the retinal activity, we found that a linear decoder (see Materials and Methods) could reach a satisfying performance, confirming previous results (Warland et al., 1997 and Marre et al., 2015). Several works have shown that it was challenging to outperform linear decoding on this task in the retina (Warland et al., 1997 and Marre et al., 2015). From this result, we can wonder whether the linear decoder is optimal, i.e., makes use of all the information present in the retinal activity, or whether this decoder is suboptimal and could be outperformed by a nonlinear decoder. To answer this question, we need to determine an upper bound on the decoding performance reachable by any decoding method. For an encoding model, the lack of reliability of the response sets an upper bound on the encoding model performance, but finding a similar upper bound for decoding is an open challenge. Here, we show that our local model can define such an upper bound.

**Figure 6.**
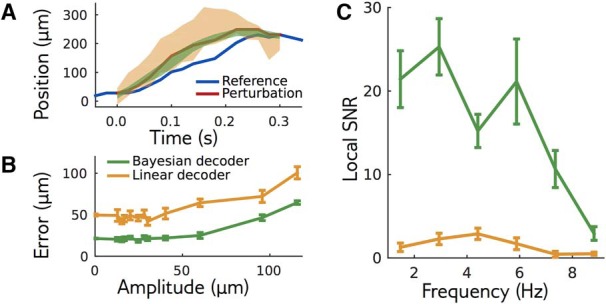
Bayesian decoding of the local model outperforms the linear decoder. ***A***, Responses to a perturbation of the reference stimulus (reference in blue, perturbation in red) are decoded using the local model (green) or a linear decoder (orange). For each decoder, the area shows one standard deviation from the mean. ***B***, Decoding error as a function of amplitude, for an example perturbation shape. ***C***, LSNR for perturbations with different frequencies (differing from the standard SNR definition to deal with locality in stimulus space and in time; Materials And Methods/Local signal to noise ratio in decoding). The performance of both decoders decreases for high frequency stimuli.

The local model is an encoding model: it predicts the probability of responses given a stimulus. Yet it can be used to create a “Bayesian decoder” using Bayesian inversion (see Materials and Methods): given a response, what is the most likely stimulus that generated this response under the model? Since the local model predicts the retinal response accurately, doing Bayesian inversion of this model should be the best decoding strategy, meaning that other decoders should perform equally or worse. When decoding the bar trajectory, we found that the Bayesian decoder was more precise than the linear decoder, as measured by the variance of the reconstructed stimulus (Fig. 6*A*). The Bayesian decoder had a smaller error than the linear decoder when decoding perturbations of small amplitudes (Fig. 6*B*). For larger amplitudes, where the local model is expected to break down, the performance of the Bayesian decoder decreased.

To quantify decoding performance as a function of the stimulus temporal frequency, we estimated a “LSNR” of the decoding signal for small perturbations of various frequencies (see Materials and Methods). The definition of the LSNR differs from the usual frequency-dependent SNR, as it is defined to deal with signals that are local in stimulus space and in time, i.e., with no invariance to time translations. We verified however that the two are equivalent when time-translation invariance is satisfied (see Discussion, Mathematical derivations). The Bayesian decoder had a much higher LSNR than the linear decoder at all frequencies (Fig. 6*C*), even if both did fairly poorly at high frequencies. This shows that, despite its good performance, linear decoding misses some information about the stimulus present in the retinal activity. This result suggests that inverting the local model, although it does not provide an alternative decoder generalizable to all possible trajectories, sets a gold standard for decoding, and can be used to test whether other decoders miss a significant part of the information present in the neural activity. It also confirms that the local model is an accurate description of the retinal response to small enough perturbations around the reference stimulus.

### Signature of efficient coding in the sensitivity

**Figure 7.**
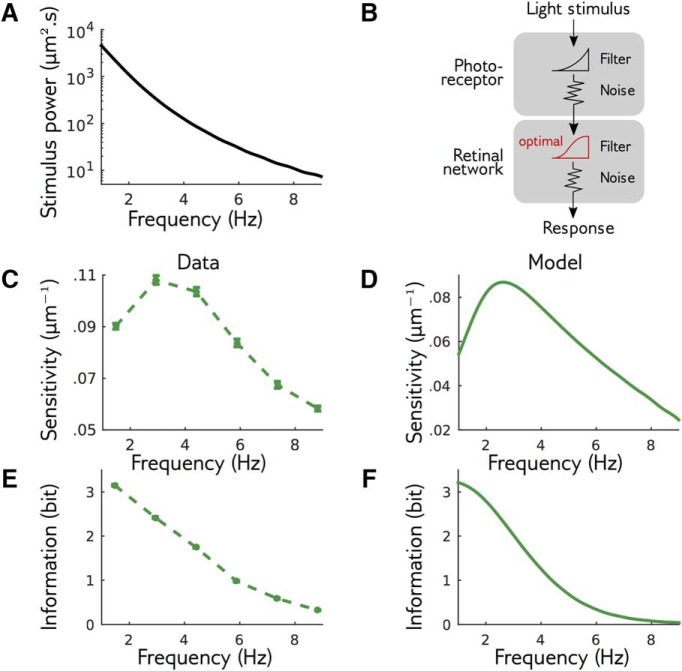
Signature of efficient coding in the sensitivity. ***A***, Spectral density of the stimulus used in experiments, which is monotonically decreasing. ***B***, Simple theory of retinal function: the stimulus is filtered by noisy photoreceptors, whose signal is then filtered by the noisy retinal network. The retinal network filter was optimized to maximize information transfer at constant output power. ***C***, Sensitivity of the recorded retina to perturbations of different frequencies. Note the nonmonotonic behavior. ***D***, Same as ***C***, but for the theory of optimal processing. ***E***, Information transmitted by the retina on the perturbations at different amplitudes. ***F***, Same as ***E***, but for the theory.

The structure of the Fisher information matrix shows that the retinal population is more sensitive to some directions of the stimulus space than others. Are these differences in the sensitivity optimal for efficient information transmission? We hypothesized that the retinal sensitivity has adapted to the statistics of the bar motion presented throughout the experiment to best transmit information about its position. Figure 7*A* represents the power spectrum of the bar motion, which is maximum at low frequencies, and quickly decays at large frequencies. We used our measure of the Fisher matrix to estimate the retinal sensitivity power as the sensitivity coefficient *c* to oscillatory perturbations as a function of temporal frequency (see Materials and Methods). Unlike the power spectrum, which depends monotonously on frequency, we found that the sensitivity is bell shaped, with a peak in frequency around 4Hz (Fig. 7*C*).

To interpret this peak in sensitivity, we studied a minimal theory of retinal function, similar to Van Hateren (1992), to test how maximizing information transmission would reflect on the sensitivity of the retinal response. In this theory, the stimulus is first passed through a low-pass filter, then corrupted by an input white noise. This first stage describes filtering due to the photoreceptors (Ruderman and Bialek, 1992). The photoreceptor output is then transformed by a transfer function and corrupted by a second external white noise, which mimics the subsequent stages of retinal processing leading to ganglion cell activity. Here the output is reduced to a single continuous signal (Fig. 7*B*; for details, see Discussion, Mathematical derivations). Note that this theory is linear: we are not describing the response of the retina to any stimulus, which would be highly nonlinear, but rather its linearized response to perturbations around a given stimulus, as in our experimental approach. To apply the efficient coding hypothesis, we assumed that the photoreceptor filter is fixed, and we maximized the transmitted information, measured by Shannon’s mutual information, over the transfer function (see Discussion, Mathematical derivations; Eq. 31). We constrained the variance of the output to be constant, corresponding to a metabolic constraint on the firing rate of ganglion cells. In this simple and classical setting, this optimal transfer function, and the corresponding sensitivity, can be calculated analytically. Although the power spectrum of the stimulus and photoreceptor output are monotonically decreasing, and the noise spectrum is flat, we found that the optimal sensitivity of the theory is bell shaped (Fig. 7*E*), in agreement with our experimental findings (Fig. 7*C*). Recall that in our reasoning, we assumed that the network optimizes information transmission for the statistics of the stimulus used in the experiment. Alternatively, it is possible that the retinal network optimizes information transmission of natural stimuli, which may have slightly different statistics. We also tested our model with natural temporal statistics (power spectrum ∼1∕*ν* ^2^ as a function of frequency *ν*; Dong and Atick, 1995) and found the same results (data not shown).

One can intuitively understand our result that a bell-shaped sensitivity is desirable from a coding perspective. On one hand, in the small frequency regime, sensitivity increases with frequency, i.e., decreases with stimulus power. This result is classic: when the input noise is small compared to stimulus, the best coding strategy for maximizing information is to whiten the input signal to obtain a flat output spectrum, which is obtained by having the squared sensitivity be inversely proportional to the stimulus power (Rieke et al., 1996; Wei and Stocker, 2016). On the other hand, at high frequencies, the input noise is too high (relative to the stimulus power) for the stimulus to be recovered. Allocating sensitivity and output power to those frequencies is therefore a waste of resources, as it is devoted to amplifying noise, and sensitivity should remain low to maximize information. A peak of sensitivity is thus found between the high SNR region, where stimulus dominates noise and whitening is the best strategy, and the low LSNR region, where information is lost into the noise and coding resources should be scarce. A result of this optimization is that the information transferred should monotonically decrease with frequency, just as the input power spectrum does (Fig. 7*F*). We tested if this prediction was verified in the data. We estimated similarly the information rate against frequency in our data, and found that it was also decreasing monotonically (Fig. 7*D*). The retinal response has therefore organized its sensitivity across frequencies in a manner that is consistent with an optimization of information transmission across the retinal network.

## Discussion

We have developed an approach to characterize experimentally the sensitivity of a sensory network to changes in the stimulus. Our general purpose was to determine which dimensions of the stimulus space most affect the response of a population of neurons, and which ones leave it invariant, a key issue to characterize the selectivity of a neural network to sensory stimuli. We developed a local model to predict how recorded neurons responded to perturbations around a defined stimulus. With this local model we could estimate the sensitivity of the recorded network to changes of the stimulus along several dimensions. We then used this estimation of network sensitivity to show that it can help define an upper bound on the performance of decoders of neural activity. We also showed that the estimated sensitivity was in agreement with the prediction from efficient coding theory.

Our approach can be used to test how optimal different decoding methods are. In our case, we found that linear decoding, despite its very good performance, was far from the performance of the Bayesian inversion of our local model, and therefore far from optimal. This result implies that there should exist nonlinear decoding methods that outperform linear decoding (Botella-Soler et al., 2016). Testing the optimality of the decoding method is crucial for brain machine interfaces (Gilja et al., 2012): in this case, an optimal decoder is necessary to avoid missing a significant amount of information. Building our local model is a good strategy for benchmarking different decoding methods.

In the retina, efficient coding theory had led to key predictions about the shape of the receptive fields, explaining their spatial extent (Atick, 1992; Borghuis et al., 2008), or the details of the overlap between cells of the same type (Liu et al., 2009; Karklin and Simoncelli, 2011; Doi et al., 2012). However, when stimulated with complex stimuli like a fine-grained image, or irregular temporal dynamics, the retina exhibits a nonlinear behavior (Gollisch and Meister, 2010). For this reason, up to now, there was no prediction of the efficient theory for these complex stimuli. Our approach circumvents this barrier, and shows that the sensitivity of the retinal response is compatible with efficient coding. Future works could use a similar approach with more complex perturbations added on top of natural scenes to characterize the sensitivity to natural stimuli.

More generally, different versions of the efficient coding theory have been proposed to explain the organization of several areas of the visual system (Dan et al., 1996; Olshausen and Field, 1996; Bell and Sejnowski, 1997; Bialek et al., 2006; Karklin and Simoncelli, 2011) and elsewhere (Machens et al., 2001; Chechik et al., 2006; Smith and Lewicki, 2006; Kostal et al., 2008). Estimating Fisher information using a local model could be used in other sensory structures to test the validity of these hypotheses.

Finally, the estimation of the sensitivity along several dimensions of the stimulus perturbations allows us to define which changes of the stimulus evoke the strongest change in the sensory network, and which ones should not make a big difference. Similar measures could in principle be performed at the perceptual level, where some pairs of stimuli are perceptually indistinguishable, while others are well discriminated. Comparing the sensitivity of a sensory network to the sensitivity measured at the perceptual level could be a promising way to relate neural activity and perception.

## Mathematical derivations

### A Derivation of discrimination coefficient in arbitrary dimension

Here, we derive Equation 5 in detail. Recall that $L_{\text{ref}}$ is a random variable taking value L(R)=ln[P(R|S)/P(R|ref)] on presentation of the reference stimulus and $L_{S}$ the random variable taking value L(R) when **R** is a response to the presentation of **S**. Then their averages are given by:

(15) $$〈L_{S}〉=\sum_{R}P(R|S)[\ln P(R|S)-\ln P(R|\text{ref})]$$

(16) $$〈L_{\text{ref}}〉=\sum_{R}P(R|\text{ref})[\ln P(R|S)-\ln P(R|\text{ref})].$$

Expanding at small $S,\;P(R|S)\approx P(R|\text{ref})(1+\partial \ln P(R|S)/\partial S^{\text{T}}{|}_{S=0}\cdot S)$, one obtains:

(17) $$〈L_{S}〉-〈L_{\text{ref}}〉=\underset{R}{\sum }P(R|\text{ref})\left({\frac{\partial \ln P(R|S)}{\partial S^{T}}|}_{S=0}\cdot S\right)\left({\frac{\partial \ln P(R|S)}{\partial S^{T}}|}_{S=0}\cdot S\right)=S^{\text{T}}\cdot I\cdot S+O(S^{3}),$$

with

(18) $$\begin{array}{l} I=(I_{tt\prime }),I_{tt\prime }=\underset{R}{\sum }P(R|\text{ref}){\frac{\partial \ln P(R|S)}{\partial S_{t}}|}_{S=0}{\frac{\partial \ln P(R|S)}{\partial S_{t\prime }}|}_{S=0}\\ =\underset{R}{\sum }{\frac{\partial \ln P(R|S)}{\partial S_{t}}|}_{S=0}{\frac{\partial \ln P(R|S)}{\partial S_{t\prime }}|}_{S=0}\\ =\frac{\partial }{\partial S_{t}}\underset{R}{\sum }P(R|S){\frac{\partial \ln P(R|S)}{\partial S_{t\prime }}|}_{S=0}-\underset{R}{\sum }P(R|ref){\frac{\partial^{2}\ln P(R|S)}{\partial S_{t}\partial S_{t\prime }}|}_{S=0}\\ =\frac{\partial }{\partial S_{t}\partial S_{t}\prime }\underset{R}{\sum }P(R|S){|}_{S=0}-\underset{R}{\sum }P(R|ref){\frac{\partial^{2}\ln P(R|S)}{\partial S_{t}\partial S_{t\prime }}|}_{S=0}\\ =\underset{R}{\sum }P(R|ref){\frac{\partial^{2}\ln P(R|S)}{\partial S_{t}\partial S_{t\prime }}|}_{S=0}, \end{array}$$

where we have used $\sum_{R}P(R|S)=1$. Similarly, the variances of these quantities are at leading order:

(19) $$〈L_{\text{ref}}^{2}〉-{〈L_{\text{ref}}〉}^{2}\approx 〈L_{S}^{2}〉-{〈L_{S}〉}^{2}\approx 〈L_{S}^{2}〉\approx \underset{R}{\sum }P(R|\text{ref}){\left({\frac{\partial \ln P(R|S)}{\partial S^{T}}|}_{S=0}\cdot S\right)}^{2}=S^{\text{T}}\cdot I\cdot S+O(S^{3}),$$

where we have used the fact that

(20) $$〈L_{S}〉=\underset{R}{\sum }P(R|S)\left({\frac{\partial \ln P(R|S)}{\partial S^{T}}|}_{S=0}\cdot S\right)+O(S^{2})=\frac{\partial }{\partial S^{T}}{\underset{R}{\sum }P(R|S)|}_{S=0}\cdot S+O(S^{2})=O(S^{2}).$$

Next, we assume that lnP(R|S) is the sum of weakly correlated variables, meaning that its distribution can be approximated as Gaussian. Thus, the random variable $L_{S}-L_{\text{ref}}$ is also distributed as a Gaussian, with mean $\mu_{L}=S^{\text{T}}\cdot I\cdot S$ and variance $\sigma_{L}^{2}=2S^{\text{T}}\cdot I\cdot S$. The discrimination probability is the probability that $L_{S}>L_{\text{ref}}$, i.e.,

(21) $$P(L_{S}-L_{\text{ref}}>0)=\int_{0}^{\infty }\frac{dx}{\sqrt{2\pi \sigma_{L}}}e^{-{\left(x-\mu_{L}\right)}^{2}/2\sigma_{L}^{2}}=\frac{1}{2}\left(1+\text{erf}\left(\frac{\mu_{L}}{2\sigma_{L}}\right)\right)=\frac{1}{2}\left(1+\text{erf}\left(\frac{d\prime }{2}\right)\right),$$

with $d\prime \equiv \mu_{L}/\sigma_{L}=\sqrt{S^{\text{T}}\cdot I\cdot S}$.

### B Fisher and linear discrimination

There exists a mathematical relation between the Fisher information of Equation 8 and linear discrimination. The linear discrimination task described earlier can be generalized by projecting the response difference, $R_{S}-R_{\text{ref}}$, along an arbitrary direction *u*:

(22) $$\Delta x=x_{S}-x_{\text{ref}}=u^{T}\cdot (R_{S}-R_{\text{ref}}).$$

Δ*x* is again assumed to be Gaussian by virtue of the central limit theorem. We further assume that perturbations **S** are small, so that $〈R_{S}〉-〈R_{\text{ref}}〉\approx (\partial 〈R_{S}〉/\partial S)\cdot S$, and that $C_{R}$ does not depend on **S**. Calculating the mean and variance of Δ*x* under these assumption gives an explicit expression of d′ in Equation 3:

(23) $$d\prime =\frac{u^{T}\cdot \frac{\partial 〈R_{S}〉}{\partial S}\cdot S}{\sqrt{u^{T}\cdot C_{R}\cdot u}}.$$

Maximizing this expression of d′ over the direction of projection **u** yields $u=\text{const}\times C_{R}^{-1}\cdot (\partial 〈R_{S}〉/\partial S)\cdot S$ and

(24) $$d\prime =\sqrt{S^{\text{T}}\cdot I_{L}\cdot S},$$

where $I_{L}={\left(\partial 〈R_{S}〉/\partial S\right)}^{\text{T}}\cdot C_{R}^{-1}\cdot (\partial 〈R_{S}〉/\partial S)$ is the linear Fisher information (Fisher, 1936; Beck et al., 2011). This expression of the sensitivity corresponds to the best possible discrimination based on a linear projection of the response.

Within the local linear model defined above, one has $\partial 〈R_{S}〉/\partial S=F\cdot C_{R}$, and $I_{L}=F\cdot C_{R}\cdot F^{T}$, which is also equal to the true Fisher information (Eq. 8): $I=I_{L}$. Thus, if the local model (Eq. 6) is correct, discrimination by linear projection of the response is optimal and saturates the bound given by the Fisher information.

Note that the optimal direction of projection only differs from the direction we used in the experiments, $u=〈R_{S}〉-〈R_{\text{ref}}〉$, by an equalization factor $C_{R}^{-1}$. We have checked that applying that factor only improves discrimination by a few percents (data not shown).

### C Local SNR for a convolutional linear decoder

In this section, we show how the local SNR defined in Equation 13 reduces to standard expression in the simpler case of a convolution decoder ϕ in the linear regime:

(25) X^=ϕ(X)=h★X+ϵ

where ★ is the convolution symbol, *h* is a stimulus independent linear filter and ϵ a Gaussian noise of covariance $C_{\epsilon }$ and zero mean. Linearizing ϕ for $X=X_{0}+S$ as in Equation 12, we obtain

(26) S^=T·S+b+ϵ,

but now the transfer matrix $T_{bb\prime }=h(b-b\prime )$ depends only on the difference between the time-bin indices *b* and b′ When *T* is applied to an oscillating perturbation of unitary amplitude ${\hat{S}}_{b}(\nu )\equiv \exp(2\pi i\nu b\delta t)$, we have:

(27) $$T\cdot S(\nu )=\overset{˜}{h}(\nu )S(\nu )$$

where $\tilde{h}(\nu )\equiv \sum_{\tau }h(\tau )\exp(2\pi i\nu \tau \delta t)$ is the Fourier coefficient of filter *h*. As a consequence of this last property, the LSNR takes the following expression (Eq. 13):

(28) $$\text{LSNR}(S(\nu ))=S{\left(\nu \right)}^{\text{T}}\cdot T^{\text{T}}\cdot C_{\epsilon }^{-1}\cdot T\cdot S(\nu )$$

(29) $$=|\overset{˜}{h}(\nu ){|}^{2}S{\left(\nu \right)}^{\text{T}}\cdot C_{\epsilon }^{-1}\cdot S(\nu ),$$

where $|\tilde{h}(\nu ){|}^{2}$ can be interpreted as the signal power at frequency *ν* for unitary stimulus perturbation. If furthermore $C_{\epsilon ,bb\prime }\equiv 〈\epsilon_{b}\epsilon_{b\prime }〉=C_{\epsilon }(b-b\prime )$, then LSNR(S(ν)) reduces to the standard expression of SNR (Woyczynski, 2010):

(30) $$\text{LSNR}(S(\nu ))=\frac{|\overset{˜}{h}(\nu ){|}^{2}}{\overset{˜}{C}(\nu )}$$

where ${\tilde{C}}_{\epsilon }(\nu )\equiv \sum_{\tau }C_{\epsilon }(\tau )\exp(2\pi i\nu \tau \delta t)$ is the noise power at frequency *ν*.

### D frequency dependence of sensitivity and information

To analyze the behavior in frequency of the sensitivity, we compute the sensitivity index for an oscillating perturbation of unitary amplitude. We apply Equation 14 with ${\hat{S}}_{b}(\nu )\equiv \exp(2\pi i\nu b\delta t)$. to estimate the spectrum of the information rate we compute its behavior within the linear theory (Van Hateren, 1992):

(31) $$\text{MI}(\nu )=\frac{1}{2}\ln\left[1+C_{S}(\nu )I(\nu )/\delta t^{2}\right]$$

where $C_{S}(\nu )$ is the power spectrum of the actual stimulus statistics (noisy damped oscillator), and $I(\nu )=(\delta t/L){\hat{S}}^{\text{T}}(\nu )\cdot I\cdot \hat{S}(\nu )$. Note that this decomposition in frequency of the transmitted information is valid because the system is linear and the stimulus is Gaussian distributed (Bernardi and Lindner, 2015).

### E efficient coding theory

To build a theory of retinal sensitivity, we follow closely the approach of Van Hateren (1992). The stimulus is first linearly convolved with a filter *f*, of power F, then corrupted by an input white noise with uniform power *H*, then convolved with the linear filter *r* of the retina network of power F, and finally corrupted again by an external white noise Γ. The output power spectrum *O*(*ν*) can be expressed as a function of frequency *ν*:

(32) $$O(\nu )=(\delta tL)G(\nu )[(\delta tL)F(\nu )C_{S}(\nu )+H]+\Gamma$$

where *C_S_*(*ν*) is the power spectrum of the input. The information capacity of such a noisy input-output channel is limited by the allowed total output power $V=\sum_{\nu }O(\nu )$, which can be interpreted as a constraint on the metabolic cost. The efficient coding hypothesis consists in finding the input-output relationship *g* *, of power $G^{*}(\nu )$, that maximizes the information transmission under a constraint on the total power of the output. The optimal Fisher information matrix can be computed in the frequency domain as:

(33) $$I(\nu )=\frac{\delta t^{4}L^{2}G^{*}(\nu )F(\nu )}{\Gamma +L\delta tG^{*}(\nu )H}.$$

The photoreceptor filter (Warland et al., 1997) was taken to be exponentially decaying in time, $f=\tau^{-1}\exp(-t/\tau )$ (for *t* ≥ 0), with *τ* = 100 ms. The curve *I*(*ν*) only depends on *H*, Γ, and *V* through two independent parameters. For the plots in Figure 7, we chose: *H* = 3.38 μm^2^/s, $\Gamma =0.02\;{\text{spikes}}^{2}s$ and $V=307\;{\text{spikes}}^{2}s,\;\delta t=20\;ms$, and *L* = 2, 500. In Figure 7*D*, we plot the sensitivity to oscillating perturbation with fixed frequency *ν*, which results in $\sqrt{I(\nu )L/\delta t}$. In Figure 7*E*, we plot the spectral density of the transferred information rate:

(34) $$\text{MI}(\nu )=\frac{1}{2}\ln\left[1+\frac{{\left(\delta tL\right)}^{2}G(\nu )F(\nu )C_{S}(\nu )}{\Gamma +(\delta tL)G(\nu )H}\right].$$

## Synthesis

Reviewing Editor: Leonard Maler, University of Ottawa Decisions are customarily a result of the Reviewing Editor and the peer reviewers coming together and discussing their recommendations until a consensus is reached. When revisions are invited, a fact-based synthesis statement explaining their decision and outlining what is needed to prepare a revision will be listed below. The following reviewer(s) agreed to reveal their identity: Richard Naud.

Editorial comment

Both reviewers have now re-reviewed the revised Ms. The reviews were very carefully done and so I have given them in full below.

The first reviewer is mostly satisfied with the changes made. If the authors carefully respond to the few additional points made by this reviewer, I will not have to consult with reviewer #1 again.

The second reviewer still had some serious concerns. With respect to presentation, this reviewer noted that the Ms oversold the results and that the conclusions should stick more closely to the actual results.

This reviewer had several critical points re the results. One important request for clarification of “about the meaning of a perturbation around a strong time-dependent stimulus and what it may tell us about the visual system”

In addition, there were problems with the mathematical presentation. Specifically, this reviewer believes that a typical reader will find a much more intuitive understanding if temporal integrals are used vs the matrix representations. I have now carefully re-read the Ms and I do agree.

Please closely follow the recommendations of the second reviewer in the revision of this Ms.

Reviewer #1

The authors made a careful review of the manuscript, responding to all my comments and questions. Now although I still do not fully abide to the premises, and there are no new neuroscience finding, I think the readers of eneuro will appreciate this novel methodology and the careful (and novel) quantitative treatment that has been developed in this article. I can now recommend the article for publication once the inaccuracies outlined below are corrected. 

1) The authors mention that efficient coding theory states that sensitivity should follow inverse input power. This is inaccurate; efficient coding theory maintains this prediction only for the case of small noise. I would argue that this fact is classical since it was described in detailed in the Rieke et al. book (1996). See also Pozzorini, et al. Nature Neuroscience (2013) Supps. for a succinct distinction between the noise-shaping regime and the whitening regime. Therefore, I disagree with the narrative starting from the fact the bell-shaped results are not explained by classical efficient coding. But it is still nice to see this transition happen, it would not be easy to obtain without the closed-loop setup. 

2) In the same section, one should state that the rationale assumes Natural PSD = Input PSD. At the moment it is mentioned in Line 456, but should be mentioned around Line 433. Similarly, the statement concerning Dong and Atick (1995) in Line 495 is odd as the spectra are not of the same quantity in Dong and Atick. It would serve as a nice justification for the assumption Natural PSD = Input PSD. 

3) The wording ‘input noise is negligible’ and ‘input noise is too high’ is misleading. It is as thought the noise has different power. Need to add w.r.t. stimulus. 

4) The sentence starting with On the one hand in line 461 is incomprehensible to me. I would suggest removing it. 

5) The introduction is appreciably better now. I commend the authors for their careful work. This is more of a note than an issue, but the last sentence of the paragraph in Line 39 comes too suddenly; it would have helped me get the proper gist of the article at the onset if it would have been made clear that of all the possible ways to understand which information is extracted (penultimate sentence of the paragraph), the authors focus on the analysis of sensitivity. 

6) Line 50 should the stimuli or the responses be complex? ‘stimuli closer to natural stimuli are complex, as they trigger non-linear responses’ 

7) Line 546, there is a typo in Gaussian 

Oh and I could not find a version with embedded figures... this makes the reviewing process more difficult. 

Reviewer #2

Unfortunately, the authors have not addressed my concern about the meaning 

of a perturbation around a strong time-dependent stimulus and what it may tell us 

about the visual system. The specific setup that they have chosen is still interesting but it is not as general as claimed by the authors: it is my strong feeling that they try to oversell their results. This is not useful for anyone in the long run and I would strongly recommend that they stick to the results that they have achieved and present them as clearly as possible. Moreover, there are certain parts in this ms, that do not look coherent to me at all or that I simply do not understand. 

If the authors carefully carry out major revisions of the ms: i) improving significantly the presentation of the derivations for the main formulas and ii) removing undue claims of generality of their method, this ms may finally merit publication in eNeuro. If not then not. 

Major issues 

1. The statements in the abstract: 

“Our approach can be used to characterize experimentally the sensitivity of neural systems to external stimuli, quantify experimentally the capacity of neural networks to encode sensory information, and relate their activity to behaviour.” 

and in the significance statement 

“Our approach is general and could be used in many sensory systems to characterize and understand their sensitivity to stimuli.” 

are not substantiated in the paper and should be removed! What the authors have done is 

to measure the response to a small perturbation *around* a certain class of time-dependent stimuli with very specific boundary conditions (no change at the beginning and the end of the window). The response depends a lot on the specific setup (statistics of reference stimulus, kind of perturbation) and is, according to my impression, *not* general at all. 

2. The authors have not followed my recommendation to reformulate their formulas in terms of temporal integrals and filters. It may be that the vector and matrix notation is very convenient for them (it is what is done, whenever integrals are evaluated numerically anyway) but it is much less transparent at least for me and I suspect for a part of the readership. It does not help that the authors added remarks that certain products can be expressed in terms of integrals. 

3. A good part of the mathematical presentation is still pretty incomprehensible to me. 

For instance, in deriving eq.(5), the authors introduce the log-ratio 

L = ln[P(R|S)/P(R|ref)] (*) 

which is, by definition through probability densities, a deterministic quantity. 

Based on (*), the authors introduce L_ref and L_S without clearly explaining how the index would affect the quantity L defined just before: What is the argument in the function L that is replaced by the index? For instance, for L_ref: is S replaced by ref? Then the ratio would be zero immediately and the following treatment would not make much sense. 

I guess the authors intended to say that e.g. L_S=ln(P(R_i|S)) and L_ref=ln(P(R_i|ref)) (not ratios!) with R_i being a specific response (turning L_S and L_ref into stochastic quantities), but this is not stated at all by the authors. Furthermore, it is unclear how t and t’ in eq.(4) are chosen, why we sum (and do not integrate) over R etc. Maybe all of this is obvious textbook knowledge, in particular, how to arrive at eq.(4) but then the textbook should be cited. However, eq.(4) also leads to eq.(5), which is stated to be one main result - so it appears unlikely that everything here is obvious. 

4. Linear decoding 

It is not clear to me what is done here. Before S(t) was the perturbation around a known reference trajectory; now, S(t) is referred to as ‘the trajectory’ (not a perturbation anymore) and it is stated explicitly that the linear decoder ‘has no information about ... the reference stimulation and its perturbation.’ For once, it is difficult to understand how a decoder could ‘have information’. More importantly, the performance in transmitting information about a whole trajectory by linear means must be necessarily worse than the performance in transmitting information about a small perturbation around a given stimulus (in the local model S(t) denotes the perturbation around the reference stimulus). So it appear (maybe wrongly so) that the authors compare a decoding of different kinds of signal, which would then not be a fair comparison. Of course, this concerns all the conclusions about the ‘gold standard’ of their method drawn by the authors. 

Details and minor issues 

l.37 by a change of along that feature dimension 

->by a change along that feature dimension. 

l. 42 that sensory structures process stimuli is a highly non-linear fashion. 

-> that sensory structures process stimuli in a highly non-linear fashion. 

caption Fig.1 ‘were combinations of simple two Fourier components’ - ‘simply’ ?? 

l.111/112 ... we searched for the smallest amplitude that will still evoke a detectable change in the retinal response. 

What is the authors’ criterion for a ‘detectable change’? Give a reference to the criterion used below. 

l.136 ‘N = 256 electrodes, or the N = 60 cells’ Why do the authors use the same variable name for two different numbers? 

149 ‘... is a projection in our joint index notation, but it can be decomposed in a summation over cells i of a time integral of the response along consecutive time-bins b’ 

What follows is not a time integral but a sum. 

l.160 ff 

“The response to the stimulation RS is noisy, making Δx the sum of many random variables (corresponding to each neuron and time bin combinations), and we can apply the central limit theorem to approximate its distribution as Gaussian, for a given perturbation at a given amplitude.” 

Again an imprecise conclusion. The sum of many random variables does not have to be Gaussian: if sufficiently correlated (maybe just in a nonlinear fashion), it is not Gaussian. Hence, the authors should verify the Gaussianity of the distribution and 

(if they can verify it) state it as an experimental observation. 

l.164 2*sigma^2 = Var(xS)+Var(xref ) 

Typically, sigma^2 is a typical parameter name for a variance (not 2*sigma^2). 

So, var(Delta_x)=Var(xS)+Var(xref ) [if xS and xref are approximately uncorrelated]. 

Maybe related: in eq.(3) I think it should be d'/sqrt(2) but not d'/2 in the error function. 

l.172 As the authors define the log ratio (L without an index), I don't understand their definition of L_ref and L_S. By definition, L_ref=0 (here replacing S by reference stimulus) and L_S is exactly L itself. More in detail: 

The authors write 

... the log-ratio L = ln[P(R|S)/P(R|ref)]. Let us call L_ref the value of L upon presentation of the reference stimulus ... 

which means that we have to replace S by ref in the just defined log-ratio? Similarly unclear for L_S. 

l.172 and eqs.(4) and (6): the authors use sometimes ‘ln’ sometimes ‘log’ and it is not clear whether they are supposed to mean the same function. 

l.180 “Estimating the Fisher Information Matrix requires building a model that can predict how the retina responds to small perturbations of the reference stimulus.” 

As far as I have understood, the Fisher information is given in terms of conditional probability functions that can be measured in experiment. Hence, it is unclear why we need a model. The authors replied to a similar remark from the first reviewer that it is related to limited data. This should be mentioned here. 

eq.(6) The notation is not clear: is S on the lhs to be taken at the current time instant t, the summation is one over t’ and the (causal) filter is taken at t-t'? 

eq.(7) Here the authors sum over time bins b and neurons i, which implies that there are no so-called noise correlations (cross-correlations among neurons upon frozen stimulus) nor temporal correlations in the fluctuations around the mean response. Both assumptions seem to be highly questionable but can be and should be checked in the experimental data. Put differently, the authors should report the values of the covariance matrix that they assume to be diagonal. 

l.208 The linear decoder 

As outlined above it is not clear what S(t) refers to: before it was the perturbation of a reference trajectory, now it is apparently the entire trajectory, which is apparently much harder to encode and decode than just a small perturbation around the reference stimulus. Clarify! 

l.222 “P(S) is taken to be the distribution of trajectories from an overdamped stochastic oscillator with the same parameters as in the experiment.” 

Do the authors mean the distribution at the sequence of discrete time instances, which would be a multivariate probability density function? What would be the expression for this density then? Or do the authors refer more generally to the path-integral expression?

l. 242,244 What is delta t in the expression for S_t? 

P is used both for the perturbation as well as for probabilities. Replace everywhere the shape by S/A to avoid this ambiguity (and to reduce the number of symbols). 

l.328 “Generalizing the result of Seung and Sompolinsky (1993) to arbitrary dimension and under the assumptions of the central limit theorem, we show that the sensitivity can be expressed in matrix form ...” 

The paper by Seung and Sompolinsky is not explained and not even mentioned in the methods section and it is not explained how it is generalized in the present ms. 

l.429 ff “In many theories of efficient coding, sensitivity is expected to follow an inverse relationship with the stimulus power (Brunel and Nadal 1998, Wei and Stocker 2016).” 

Where in the paper by Brunel and Nadal is the sensitivity related to the power spectrum (in a frequency-resolved manner)?

## Author Response

Summary of changes

“Synthesis Statement for Author (Required):

Editorial comment

Both reviewers have now re-reviewed the revised Ms. The reviews were very carefully done and so I have given them in full below.

The first reviewer is mostly satisfied with the changes made. If the authors carefully respond to the few additional points made by this reviewer, I will not have to consult with reviewer #1 again.

We have fully addressed the (mostly minor) comments of the first reviewer.

The second reviewer still had some serious concerns. With respect to presentation, this reviewer noted that the Ms oversold the results and that the conclusions should stick more closely to the actual results.

The main point of contention is that our method allows us to estimate the sensitivity of sensory systems locally, around a stimulus of choice, and does not allow to estimate sensitivity over the entire stimulus space. We have now added that clarification in the sentences quoted by the reviewer, as well as an additional clarification sentence in the introduction. However, we believe it is important to stress that our approach, far from being restricted to the retina, can be transposed to other systems, and maintain our claim of generality.

This reviewer had several critical points re the results. One important request for clarification of “about the meaning of a perturbation around a strong time-dependent stimulus and what it may tell us about the visual system””

We have responded to that point (see below), and changed the text accordingly to explain our argument.

“In addition, there were problems with the mathematical presentation. Specifically, this reviewer believes that a typical reader will find a much more intuitive understanding if temporal integrals are used vs the matrix representations. I have now carefully re-read the Ms and I do agree.

Please closely follow the recommendations of the second reviewer in the revision of this Ms.”

We have followed the reviewer's recommendations regarding the mathematical presentation as well as we could, including the temporal integral representation. Some confusion seems to have come from the fact that it was not always clear to the reviewer what was a vector and what was a scalar (single number). We have now marked all our vectors and matrices with bold fonts (as is conventionally done in many fields).

“Reviewer #1

The authors made a careful review of the manuscript, responding to all my comments and questions. Now although I still do not fully abide to the premises, and there are no new neuroscience finding, I think the readers of eneuro will appreciate this novel methodology and the careful (and novel) quantitative treatment that has been developed in this article. I can now recommend the article for publication once the inaccuracies outlined below are corrected. “

We thank the reviewer for acknowledging the strengths of the paper and appreciating the effort made in the previous revision.

“1) The authors mention that efficient coding theory states that sensitivity should follow inverse input power. This is inaccurate; efficient coding theory maintains this prediction only for the case of small noise. I would argue that this fact is classical since it was described in detailed in the Rieke et al. book (1996). See also Pozzorini, et al. Nature Neuroscience (2013) Supps. for a succinct distinction between the noise-shaping regime and the whitening regime. Therefore, I disagree with the narrative starting from the fact the bell-shaped results are not explained by classical efficient coding. But it is still nice to see this transition happen, it would not be easy to obtain without the closed-loop setup. “

We agree with the reviewer that viewing whitening as the cornerstone of efficient coding is too restrictive.

We have rephrased this part to remove the narrative questioned by the reviewer. In particular, we have remove the phrase "contrary to classical prediction", and the sentence "In the absence of input noise, many theories of efficient coding predict that sensitivity should follow an inverse relationship with the stimulus power \citep{Brunel1998,Wei2016}, thus whitening the output signal". The interpretation in terms of whitening is still there, later in the text, and we now cite Rieke et al. 1996.

“2) In the same section, one should state that the rationale assumes Natural PSD = Input PSD. At the moment it is mentioned in Line 456, but should be mentioned around Line 433. Similarly, the statement concerning Dong and Atick (1995) in Line 495 is odd as the spectra are not of the same quantity in Dong and Atick. It would serve as a nice justification for the assumption Natural PSD = Input PSD. “

The reviewer is right that this needs better explaining. We in fact explore two alternative versions of efficient coding. In the first one, which is the one we focus on here, the retinal response has adapted to the statistics of the bar. We then test efficient coding by taking the input PSD to solve the information optimisation problem. The second version of efficient coding is that the retina is optimal for natural statistics, and does not necessarily adapt to those of the experiment. Our bar motion is fairly close to natural statistics, but to check that our results would still hold in this second version, we explicitly replaced the moving-bar power spectrum used in the experiment with what is believed to be the typical power spectrum of natural time series, namely 1/nu^2. Doing so did not affect our results much, in particular the bell shape of the sensitivity curve.

We have added the sentence: "We hypothesized that the retinal sensitivity has adapted to the statistics of the bar motion presented throughout the experiment to best transmit information about its position" before discussing the efficient coding calculation (version 1 of efficient coding). We then explain how we test the theory again on a natural-like 1/nu^2 power spectrum (version 2):

"Recall that in our reasoning, we assumed that the network optimizes information transmission for the statistics of the stimulus used in the experiment. Alternatively, it is possible that the retinal network optimizes information transmission of natural stimuli, which may have slightly different statistics. We also tested our model with natural temporal statistics (power spectrum $\sim 1/\nu^2$ as a function of frequency $\nu$, \cite{Dong1995}) and found the same results (data not shown)."

We hope that with these explanations our approach is now clearer.

“3) The wording ‘input noise is negligible’ and ‘input noise is too high’ is misleading. It is as thought the noise has different power. Need to add w.r.t. stimulus. “

The reviewer is right that the noise magnitude must be compared to signal.

We have changed the sentences by adding "compared to the stimulus" and "relative to the stimulus".

“4) The sentence starting with On the one hand in line 461 is incomprehensible to me. I would suggest removing it. “

We agree the sentence was not clear.

We have replaced the sentence by:

"On one hand, in the small frequency regime, sensitivity increases with frequency, i.e. decreases with stimulus power."

“5) The introduction is appreciably better now. I commend the authors for their careful work. This is more of a note than an issue, but the last sentence of the paragraph in Line 39 comes too suddenly; it would have helped me get the proper gist of the article at the onset if it would have been made clear that of all the possible ways to understand which information is extracted (penultimate sentence of the paragraph), the authors focus on the analysis of sensitivity. “

We thank the reviewer for appreciating our effort in rewriting the introduction.

We have reformulated the first paragraph to address the reviewer's comment, by mentioning sensitivity earlier (in the second sentence), and removing the incriminated sentence at the end. The second sentence now reads:

"A crucial step towards understanding the sensory processing performed by a given brain area is to characterize its sensitivity \citep{Benichoux2017}, by determining which features of the sensory input are coded in the activity of these sensory neurons, and which features are discarded."

“6) Line 50 should the stimuli or the responses be complex? ‘stimuli closer to natural stimuli are complex, as they trigger non-linear responses’ “

We have changed the text by replacing "complex" by "harder to characterize".

“7) Line 546, there is a typo in Gaussian “

Thanks for catching this. We have corrected the typo.

“Oh and I could not find a version with embedded figures... this makes the reviewing process more difficult. “

We apologize for this, but the eNeuro submission system requirements makes it impossible to provide a full pdf with the embedded figures. Or rather, such a version is requested by the system, but is not shared with the reviewers because it is merged with the title page containing the author list. However we will try and attach such a PDF with embedded figures as a "companion paper" in the next revision.

“Reviewer #2

Unfortunately, the authors have not addressed my concern about the meaning

of a perturbation around a strong time-dependent stimulus and what it may tell us

about the visual system.”

In the previous revision, we had extensively rewritten the introduction to explain our approach better. The main point is that characterizing the set of features that the retina (or any sensory system) is sensitive to is key to understanding how it processes information. Because of the retina's many nonlinearities, this sensitivity characterization depends on where one is in stimulus space. When there is no model that can characterize the response to arbitrary stimuli, a local approach is required. At the same time, we agree with the reviewer that the fact that the approach is local limits its ability to generalize to other parts of the stimulus space. However, some general insights can be obtained that are robust to the choice of reference stimulus. This is what we do in this paper, where we studied two reference stimuli. Our result of efficient coding holds regardless of the choice of reference stimulus.

We have added in the introduction that the characterization is local:

"Here we present a novel approach to measure experimentally the local sensitivity of a non-linear network."

We have also stated more explicitly that the local model does not generalize to arbitrary stimuli, and explain why it is still interesting to get local models, as these models may share common and robust features (such as efficient coding):

"Although this characterization is necessarily local and does not generalize to the entire stimulus space, one can hope to use it to reveal general principles that are robust to the chosen reference stimulus."

“The specific setup that they have chosen is still interesting but it is not as general as claimed by the authors: it is my strong feeling that they try to oversell their results. This is not useful for anyone in the long run and I would strongly recommend that they stick to the results that they have achieved and present them as clearly as possible. Moreover, there are certain parts in this ms, that do not look coherent to me at all or that I simply do not understand.

If the authors carefully carry out major revisions of the ms: i) improving significantly the presentation of the derivations for the main formulas and ii) removing undue claims of generality of their method, this ms may finally merit publication in eNeuro. If not then not.

Major issues

1. The statements in the abstract:

“Our approach can be used to characterize experimentally the sensitivity of neural systems to external stimuli, quantify experimentally the capacity of neural networks to encode sensory information, and relate their activity to behaviour.”

and in the significance statement

“Our approach is general and could be used in many sensory systems to characterize and understand their sensitivity to stimuli.”

are not substantiated in the paper and should be removed! What the authors have done is

to measure the response to a small perturbation *around* a certain class of time-dependent stimuli with very specific boundary conditions (no change at the beginning and the end of the window). The response depends a lot on the specific setup (statistics of reference stimulus, kind of perturbation) and is, according to my impression, *not* general at all. “

We respectfully disagree with the reviewer that our method is not general. The purpose of these sentences is to make it clear that the method can be used outside the retinal context. Of course it is illustrated on a particular system with a particular choice of stimulus, but the point is that the approach can be transposed to other contexts. However, the reviewer is correct that this characterization is local and that this should be made clear: the sensitivity estimated locally around a given reference stimulus does not allow us to estimate the sensitivity of the system around other reference stimuli.

To emphasize the local aspect of our analysis in the context of other applications, we have added the word "locally" in these sentences.

“2. The authors have not followed my recommendation to reformulate their formulas in terms of temporal integrals and filters. It may be that the vector and matrix notation is very convenient for them (it is what is done, whenever integrals are evaluated numerically anyway) but it is much less transparent at least for me and I suspect for a part of the readership. It does not help that the authors added remarks that certain products can be expressed in terms of integrals. “

We now give a formulation in terms in integrals as well, and added the necessary extra explanations and notations necessary for this new formulation.

“3. A good part of the mathematical presentation is still pretty incomprehensible to me.

For instance, in deriving eq.(5), the authors introduce the log-ratio

L = ln[P(R|S)/P(R|ref)] (*)

which is, by definition through probability densities, a deterministic quantity.

Based on (*), the authors introduce L_ref and L_S without clearly explaining how the index would affect the quantity L defined just before: What is the argument in the function L that is replaced by the index? For instance, for L_ref: is S replaced by ref? Then the ratio would be zero immediately and the following treatment would not make much sense.

I guess the authors intended to say that e.g. L_S=ln(P(R_i|S)) and L_ref=ln(P(R_i|ref)) (not ratios!) with R_i being a specific response (turning L_S and L_ref into stochastic quantities), but this is not stated at all by the authors. Furthermore, it is unclear how t and t’ in eq.(4) are chosen, why we sum (and do not integrate) over R etc. Maybe all of this is obvious textbook knowledge, in particular, how to arrive at eq.(4) but then the textbook should be cited. However, eq.(4) also leads to eq.(5), which is stated to be one main result - so it appears unlikely that everything here is obvious. “

L is not a deterministic quantity, because it depends on the response R, which is itself stochastic, and depends on what stimulus was shown. L_ref and L_S are two random variables that take value L(R) when R is drawn from the responses to the reference and perturbed stimuli, respectively. We agree that this part was not clear, and we think this point is the main source of confusion for the reviewer.

The sum in the definition of the Fisher information is a sum and not an integral because the response is discrete in our framework. Equation 4 is a general definition of the matrix elements of the matrix I for each t,t' pairs, so t and t' are not chosen. This is a standard way of defining matrices. The definition of the Fisher information is standard: https://en.wikipedia.org/wiki/Fisher_information

as is the definition of the sensitivity index d' from the mean and variance of a signal:

https://en.wikipedia.org/wiki/Sensitivity_index

However if the reviewer had difficulties here, it is probably useful to help the reader to follow the math by being clearer about our definitions.

We now explain the use of L for discrimination in more precise terms, and we have rewritten this part extensively.

To help with the definition of the Fisher information, we have added I = (I_tt') to make it clear that I_tt' are elements of the matrix I. The Fisher information matrix is now marked in bold (as all vectors and matrices in the revised version).

To help the reader with the mathematics of this section, we have added a reference to the textbook of MacMillan and Creeman, as well as to Seung and Sompolinsky 1993, who derive the same result with 1-dimensional stimuli (i.e. I and S being a 1x1 matrices, or scalars).

“4. Linear decoding

It is not clear to me what is done here. Before S(t) was the perturbation around a known reference trajectory; now, S(t) is referred to as ‘the trajectory’ (not a perturbation anymore)”

The reviewer is correct, the notation was inconsistent. We apologize for this notational confusion.

We now call the position of the bar X(t), and define the reference trajectory as X_0(t), so that the trajectory to decode is X(t)=X_0(t)+S(t), and we have added explanations about these new notations.

“and it is stated explicitly that the linear decoder ‘has no information about ... the reference stimulation and its perturbation.’ For once, it is difficult to understand how a decoder could ‘have information’.”

The decoder could "have information" in a Bayesian sense, by including into it some "prior" knowledge about what's being played. In this case we wanted to make it clear that the linear decoder does not include prior information about the fact that the stimulus is close to a reference trajectory. We note that we added that sentence as per reviewer request.

However, the linear decoder implicitly does use prior information, but about the statistics of the overdamped oscillator, as it was trained on those statistics. For a fair comparison we used the same prior when building our local-model-based Bayesian decoder.

We have rewritten these sentences to make it clear what prior information is included, implicitly (for the linear decoder) or explicitly (for the Bayesian decoder).

“More importantly, the performance in transmitting information about a whole trajectory by linear means must be necessarily worse than the performance in transmitting information about a small perturbation around a given stimulus (in the local model S(t) denotes the perturbation around the reference stimulus). So it appear (maybe wrongly so) that the authors compare a decoding of different kinds of signal, which would then not be a fair comparison. Of course, this concerns all the conclusions about the ‘gold standard’ of their method drawn by the authors. “

We made sure that the comparison was fair by comparing performance in reconstructing the difference between perturbation and reference (i.e. S), on the same temporal support. The two signals to be reconstructed are thus exactly the same, with the same prior information included as explained above.

“Details and minor issues

l.37 by a change of along that feature dimension

-<by a=”” change="" along="" that="" feature="" dimension.="" “<br="">

Thank you. Corrected.

“l. 42 that sensory structures process stimuli is a highly non-linear fashion.

-< that sensory structures process stimuli in a highly non-linear fashion. “

Thank you. Corrected.

“caption Fig.1 ‘were combinations of simple two Fourier components’ - ‘simply’ ?? “

We have removed the word "simple".

“l.111/112 ... we searched for the smallest amplitude that will still evoke a detectable change in the retinal response.

What is the authors’ criterion for a ‘detectable change’? Give a reference to the criterion used below. “

The criterion is the point of this whole section, and is explained in detail below.

We have now added "as we explain below", to make it clear that the precise definition is yet to come.

“l.136 ‘N = 256 electrodes, or the N = 60 cells’ Why do the authors use the same variable name for two different numbers? “

For readability and clarity we think it is clearer (and, we must stress, completely standard) to denote these quantities with the same letter, as our theoretical and computational approach applies in exactly the same way. The same goes for the response R.

We now make it clearer that the same notation is used for both contexts (electrodes and cells) for mathematical convenience.

“149 ‘... is a projection in our joint index notation, but it can be decomposed in a summation over cells i of a time integral of the response along consecutive time-bins b’

What follows is not a time integral but a sum. “

We have changed the text to "summation".

“l.160 ff

“The response to the stimulation RS is noisy, making Δx the sum of many random variables (corresponding to each neuron and time bin combinations), and we can apply the central limit theorem to approximate its distribution as Gaussian, for a given perturbation at a given amplitude."

Again an imprecise conclusion. The sum of many random variables does not have to be Gaussian: if sufficiently correlated (maybe just in a nonlinear fashion), it is not Gaussian. Hence, the authors should verify the Gaussianity of the distribution and

(if they can verify it) state it as an experimental observation. “

As argued below in response to another point, we neglect noise correlations in our framework, which makes the terms of the sum independent. In general, it should be noted that sums of even weakly correlated variables are still approximately Gaussian. The text already mentions that the distribution is "approximately" Gaussian, emphasizing that this is an approximation. The distributions in Fig. 3B show that this approximation is reasonable.

We now refer to Fig. 3B to show the actual distributions.

“l.164 2*sigma^2 = Var(xS)+Var(xref )

Typically, sigma^2 is a typical parameter name for a variance (not 2*sigma^2).

So, var(Delta_x)=Var(xS)+Var(xref ) [if xS and xref are approximately uncorrelated].

Maybe related: in eq.(3) I think it should be d'/sqrt(2) but not d'/2 in the error function. “

xS and xref both have variance sigma^2, making the sum of the two = 2 sigma^2.

We have now clarified this.

“l.172 As the authors define the log ratio (L without an index), I don't understand their definition of L_ref and L_S. By definition, L_ref=0 (here replacing S by reference stimulus) and L_S is exactly L itself. More in detail:

The authors write

... the log-ratio L = ln[P(R|S)/P(R|ref)]. Let us call L_ref the value of L upon presentation of the reference stimulus ...

which means that we have to replace S by ref in the just defined log-ratio? Similarly unclear for L_S. “

We hope that the changes made in that section (explained in response to a previous point) have now cleared the reviewer's confusion. L_ref is a stochastic variable and is not equal to zero, not even on average. Both L_ref and L_S are realizations of L(R), but with R drawn from different ensembles of responses, as explained above.

“l.172 and eqs.(4) and (6): the authors use sometimes ‘ln’ sometimes ‘log’ and it is not clear whether they are supposed to mean the same function. “

We apologize for this inconsistency.

We now call everything ln (natural log).

“l.180 “Estimating the Fisher Information Matrix requires building a model that can predict how the retina responds to small perturbations of the reference stimulus."

As far as I have understood, the Fisher information is given in terms of conditional probability functions that can be measured in experiment. Hence, it is unclear why we need a model. The authors replied to a similar remark from the first reviewer that it is related to limited data. This should be mentioned here. “

Unless one can sample the space of N*B-dimensional responses with accuracy, which is impractical for 60 neurons and 13 multiple time bins (2^(60*13) possible responses and as many frequencies to report), one does need a model.

We now make it clearer that the limitation is due to the limited sampling of the multi-dimensional response:

"Because sampling the full response probability distribution P(R|S) would require estimating 2^{N×B} numbers for each perturbation S, estimating the Fisher Information Matrix directly is impractical, and requires building a model that can predict how the retina responds to small perturbations of the reference stimulus."

“eq.(6) The notation is not clear: is S on the lhs to be taken at the current time instant t, the summation is one over t’ and the (causal) filter is taken at t-t'? “

S on the left-hand side has no index and is thus a vector (as is R), per standard convention used throughout the paper. There is not t' in that equation, so no clear sense of what the reviewer means by the second question. Note that the filter is not time-translation invariant as the reviewer seems to suggest, so it depends on both the stimulus time t, and response time (spike time t_i in the new version, or bin b).

The confusion seems to come from the fact that the reviewer interpreted S in the lhs as a number, while it is a vector. We now denote all vectors and matrices with bold faces.

“eq.(7) Here the authors sum over time bins b and neurons i, which implies that there are no so-called noise correlations (cross-correlations among neurons upon frozen stimulus) nor temporal correlations in the fluctuations around the mean response. Both assumptions seem to be highly questionable but can be and should be checked in the experimental data. Put differently, the authors should report the values of the covariance matrix that they assume to be diagonal. “

We agree with the reviewer that neglecting noise correlations is an assumption. The validation of the model in Fig. 4 and then Fig. 5 validates this assumption (and others) a posteriori. The same-time cross-correlogram between neurons below shows that noise correlations are indeed small.

We also note that including noise correlations in the model would require to learn of the order of (NxB)^2 = 608400 numbers (across neurons and time bins), certainly leading to overfitting. Adding such correlations would further require to specify a model (such as ones of maximum entropy), which are numerous, and often computationally intractable. The vast majority of work on neural populations assumes conditional independence. Going beyond that is way beyond the scope of this paper. We feel that showing additional data about noise correlations in our experiments would overburden the paper and distract the reader's attention.

“l.208 The linear decoder

As outlined above it is not clear what S(t) refers to: before it was the perturbation of a reference trajectory, now it is apparently the entire trajectory, which is apparently much harder to encode and decode than just a small perturbation around the reference stimulus. Clarify! “

We have now renamed S to X in that context (see response above).

“l.222 “P(S) is taken to be the distribution of trajectories from an overdamped stochastic oscillator with the same parameters as in the experiment."

Do the authors mean the distribution at the sequence of discrete time instances, which would be a multivariate probability density function? What would be the expression for this density then? Or do the authors refer more generally to the path-integral expression?"

By virtue of the convention that S is a vector (S_t), P(S) is the multivariate probability distribution of S_t for each time t. Because the dynamics is that of an overdamped harmonic oscillator, that distribution is a multivariate Gaussian, with parameters derived from the characteristics of the oscillator (mass, drag, harmonic frequency). Calculation details are given in earlier papers.

To make it clear what is a vector and what is a single number (scalar), we now mark all our vectors and matrices in bold fonts. We have now added a reference to an (anonymized) paper containing these derivations, which are not essential for the present paper.

“l. 242,244 What is delta t in the expression for S_t? “

delta t is defined the "Perturbation" paragraph of the Methods.

“P is used both for the perturbation as well as for probabilities. Replace everywhere the shape by S/A to avoid this ambiguity (and to reduce the number of symbols). “

We thank the reviewer for the suggestion. We prefer not to replace P by S/A, as this would make formulas harder to understand, especially the shape-dependent coefficient c(P), and remove a conceptually important distinction between perturbation shape and amplitude.

We have now renamed the perturbation shape P by Q.

“l.328 “Generalizing the result of Seung and Sompolinsky (1993) to arbitrary dimension and under the assumptions of the central limit theorem, we show that the sensitivity can be expressed in matrix form ..."

The paper by Seung and Sompolinsky is not explained and not even mentioned in the methods section and it is not explained how it is generalized in the present ms.

The result of Seung and Sompolinsky is now cited in the Methods section."

Seung and Sompolinsky showed the result of Eq. 13 for one-dimension (scalar) stimuli S. Our results generalize theirs by showing it for arbitrary dimensions.

The text now clearly states: "This result generalizes that of Seung and Sompolinsky (1993), initially derived for one-dimensional stimuli, to arbitrary dimensions."

“l.429 ff “In many theories of efficient coding, sensitivity is expected to follow an inverse relationship with the stimulus power (Brunel and Nadal 1998, Wei and Stocker 2016)."

Where in the paper by Brunel and Nadal is the sensitivity related to the power spectrum (in a frequency-resolved manner)?"

The paper in question shows a more general result about how sensitivity optimally scales with input variance. The application to frequency domain is just a consequence of their result cast in Fourier space. But we agree that finding this correspondence may not be straightforward.

We have changed to a reference to the book et Rieke et al. 1996 where whitening is discussed in the simple context of linear processing in the frequency domain.
